# The Microbiota–Gut–Brain Axis and Nutritional Interventions in Amyotrophic Lateral Sclerosis: Pathophysiological Mechanisms, Neuroinflammation, and Non-Motor Manifestations—Scoping Review

**DOI:** 10.3390/pathophysiology33030059

**Published:** 2026-08-10

**Authors:** Elena Sanchis-Sanchis, José Enrique de la Rubia Ortí, David Sancho-Cantus, Cristina Cunha-Pérez, Jorge Casaña-Mohedo

**Affiliations:** 1 Doctoral School, Catholic University of Valencia San Vicente Mártir, 46001 Valencia, Spain; elena.ss@mail.ucv.es; 2 Faculty of Medicine and Health Sciences, Catholic University of Valencia San Vicente Mártir, 46007 Valencia, Spain; joseenrique.delarubi@ucv.es (J.E.d.l.R.O.); cristina.cunha@ucv.es (C.C.-P.); jorge.casana@ucv.es (J.C.-M.); 3 Nursing and Mental Health Research Group, Department of Nursing, Catholic University of Valencia, 46007 Valencia, Spain

**Keywords:** Amyotrophic Lateral Sclerosis, pathophysiology, microbiota–gut–brain axis, intestinal permeability, neuroinflammation, psychobiotics

## Abstract

Amyotrophic Lateral Sclerosis (ALS) is a progressive neurodegenerative disorder in which systemic pathophysiological alterations significantly contribute to disease progression and non-motor manifestations, such as depression and anxiety. The microbiota–gut–brain axis represents a critical bidirectional pathway in which intestinal dysbiosis and epithelial barrier disruption catalyze central neuroinflammation. This scoping review synthesizes evidence from 43 empirical and analytical studies across 28 countries and maps the findings under the WHO International Classification of Functioning (ICF) framework. Pathophysiological data reveal a profound taxonomic shift in patients with ALS, characterized by severe depletion of neuroprotective, butyrate-producing genera (Akkermansia and Prevotella) and enrichment of pro-inflammatory Enterobacteriaceae. This dysbiotic state leads to structural damage to the intestinal mucosa, alteration of Paneth cells, and downregulation of tight junction proteins (zonulin), triggering a “leaky gut” phenomenon. Subsequent systemic translocation of lipopolysaccharides (LPS) induces TLR4-mediated endotoxemia, microglial hyperactivation, and accelerated motor neuron apoptosis. Conversely, therapeutic modulation via Fecal Microbiota Transplantation (FMT), psychobiotics, and metabolic interventions (ketogenic or Mediterranean diets) has demonstrated significant efficacy in restoring epithelial integrity, mitigating mitochondrial hypermetabolism, and reducing emotional distress. This review identifies a critical research gap in the microstructural characterization of the enteric nervous system in ALS. Incorporating microbiome-targeted biomarkers into clinical protocols is crucial for implementing a stratified, multi-systemic therapeutic strategy aimed at enhancing patient prognosis and psychological well-being.

## 1. Introduction

Amyotrophic Lateral Sclerosis (ALS) is a progressive, disabling neurodegenerative disorder with a high mortality rate [1], characterized by the selective loss of upper and lower motor neurons [2]. Although traditionally defined by its motor symptoms, it is now recognized to include non-motor manifestations, including mood alterations such as depression and anxiety [3,4]. Emotional distress, ranging from major depressive episodes and generalized anxiety to pseudobulbar affect (emotional lability) [5,6], affects a considerable proportion of patients [7]. This distress negatively influences not only their quality of life but also disease progression and survival [8]. Despite efforts to understand the etiology of these manifestations, the underlying biological mechanisms remain a subject of intense debate [9,10].

In the last decade, the study of ALS pathogenesis has shifted from the central nervous system (CNS) to systemic communication. In this context, the microbiota–gut–brain (MGB) axis has emerged as a critical modulator of neurological and emotional health [11,12]. This bidirectional signaling system integrates neural pathways (such as the vagus nerve), neuroendocrine routes, and peripheral immunological signals [13,14]. The intestinal microbiota not only regulates metabolic homeostasis but also synthesizes neurotransmitters [15] and bioactive metabolites, including short-chain fatty acids (SCFAs) [16], tryptophan precursors, and gamma-aminobutyric acid (GABA) [17], all of which possess the capacity to cross the blood-brain barrier or signal through afferent projections to brain areas involved in affective regulation, such as the prefrontal cortex and amygdala [18].

Recent translational evidence suggests that intestinal dysbiosis plays an active role in the neuroinflammation characteristic of ALS [19,20]. It has been observed that alterations in intestinal permeability (the “leaky gut” phenomenon) allow the translocation of lipopolysaccharides and other pathogen-associated molecular patterns into the systemic circulation [19,21,22,23]. This immunogenic response triggers microglial activation and the recruitment of pro-inflammatory monocytes into the CNS, exacerbating oxidative stress and neuronal apoptosis [24,25,26]. While it has been hypothesized that this chronic inflammatory state is a determining factor for psychological distress and psychiatric vulnerability in neurodegenerative diseases, specific evidence in patients with ALS is currently scattered and lacks an integrative synthesis [27,28].

This study aimed to identify and characterize the factors determining emotional distress in patients with ALS via the MGB axis through a scoping review. Due to the diverse nature of study protocols and the nascent stage of this field, a scoping review represents the most effective methodological framework to address this issue [29]. This approach facilitates mapping the extent, range, and nature of research activity in areas where evidence remains unconsolidated [30]. This approach is fundamental not only to identify the common molecular mediators between the microbiota and emotional well-being but also to highlight the methodological gaps and knowledge voids that prevent the transition toward microbiome-based clinical interventions.

## 2. Materials and Methods

### 2.1. Research Design

A scoping review was conducted following the conceptual framework proposed by Arksey and O’Malley and the recommendations of the Joanna Briggs Institute (JBI) Evidence Synthesis Manual [31,32]. This study was designed to map the influence of the microbiota–gut–brain axis on the development and modulation of emotional distress in patients with ALS. The reporting of this review adhered to the Preferred Reporting Items for Systematic Reviews and Meta-Analyses extension for Scoping Reviews (PRISMA-ScR) checklist [33]. The authors state that they have used Claude (v1.24012.1) solely for the purpose of translating texts and for typesetting and improving the texts.

The research question was formulated following the PCC structure (Table 1): What is the influence of the microbiota–gut–brain axis on the development and modulation of emotional distress in patients with ALS?

### 2.2. Protocol and Registration

The protocol for this review was defined a priori and registered on the Open Science Framework (OSF) platform [34] (https://osf.io/mbr59/(accessed on 20 June 2026), following international standards to ensure transparency and reproducibility of the methodological process. No modifications or amendments were made to the protocol after its initial registration with the OSF.

### 2.3. Search Strategy and Information Sources

Literature identification was conducted through a systematic search of the PubMed/MEDLINE, Web of Science (WoS), Scopus, and EMBASE databases, covering the period from January 2016 to April 2026. A Boolean search strategy was designed, combining MeSH terms with free-text keywords, structured around three conceptual axes: (1) the primary pathology (“Amyotrophic Lateral Sclerosis” OR “Motor Neuron Disease”), (2) the non-motor domains of interest (“Psychological Distress” OR “ Depression” OR “Anxiety”), and (3) the intestinal interaction (“Gastrointestinal Microbiome OR Gut Microbiota OR Dysbiosis”). Additionally, a manual search of the reference lists of the selected articles was performed (snowballing technique) [35] to identify key studies that may have been omitted by the database algorithms.

### 2.4. Evidence Selection Procedure

The selection process was conducted systematically and transparently to ensure replicability. In the initial phase, duplicate records were removed using bibliographic management software (Mendeley Reference Manager 2.148.0). Subsequently, two independent reviewers screened the titles and abstracts based on predefined inclusion criteria using the Rayyan web application (https://www.rayyan.ai (accessed on 15 May 2026). Articles that raised doubts or met the criteria were subjected to full-text review. At this stage, the suitability of the studies was evaluated, specifically considering the use of structural equation modeling or path analysis. Any discrepancies between the reviewers during the selection process were resolved through the intervention of a third reviewer or by consensus following a technical discussion [36,37,38].

### 2.5. Evidence Analysis and Synthesis

Given the nature of the included studies, the results were synthesized through qualitative thematic analysis supported by quantitative evidence. The findings were categorized into four critical axes: ALS biomarkers, gastrointestinal function, nutrition, and emotional distress. Due to the heterogeneity and qualitative nature of the synthesis, no quantitative sensitivity analyses or meta-regressions were performed.

### 2.6. Risk of Bias and Methodological Quality Assessment

While scoping reviews typically do not require a formal evaluation of evidence quality, as is the case with systematic reviews, this study chose to conduct such an assessment to enhance the validity of its findings. The Joanna Briggs Institute (JBI) Critical Appraisal Tool for cross-sectional and analytical studies was used [39]. This tool allows for the scoring of bias risk across critical areas, such as internal validity (whether the sample is representative of the ALS population; for instance, considering bulbar vs. spinal onset distribution), measurement validity (the use of validated psychometric scales and the precision of biomarkers), confounding factors, and statistical analysis (the appropriate identification of confounding variables such as age and sex, and the correct use of multivariate models for predictive modeling). The included studies were classified according to their risk of bias (Low, Moderate, or High) (Table S1).

## 3. Results

### 3.1. Study Selection Procedure

The primary search identified a total of 534 records. Concurrently, 7 additional articles of interest were identified through manual review or “snowballing.” After the removal of 120 duplicates, 414 unique records proceeded to the screening phase. The screening of titles and abstracts against the selection criteria resulted in the exclusion of 357 studies.

Regarding the inclusion criteria, the population was assessed by selecting research involving adults with a confirmed diagnosis of ALS, encompassing both bulbar and spinal onset. Concerning the central concept, studies were required to analyze the taxonomic composition of the microbiota or gastrointestinal functionality and link these findings to mental health variables. Likewise, studies exploring mediating factors such as dietary intake (fiber and vitamins B/C) and markers of oxidative stress were considered.

The following conditions were excluded because they could introduce significant bias in the assessment of distress or microbiome composition. We discarded: (i) studies involving patients with severe cognitive impairment or frontotemporal dementia, given the inability to ensure the validity of self-report instruments; and (ii) studies with patients presenting severe systemic comorbidities (such as inflammatory bowel disease) or substance dependence.

The remaining 57 articles from the primary search were retrieved and merged with the seven articles obtained manually, resulting in 64 studies for the eligibility phase. A detailed full-text evaluation led to the definitive exclusion of 14 additional studies, which, despite the initial perspective, only addressed the core dietary or psychological interventions of this review in a residual or insufficient manner. Consequently, this scoping review was consolidated based on a robust final sample of 43 empirical and analytical studies (Table S1).

**Figure 1:**
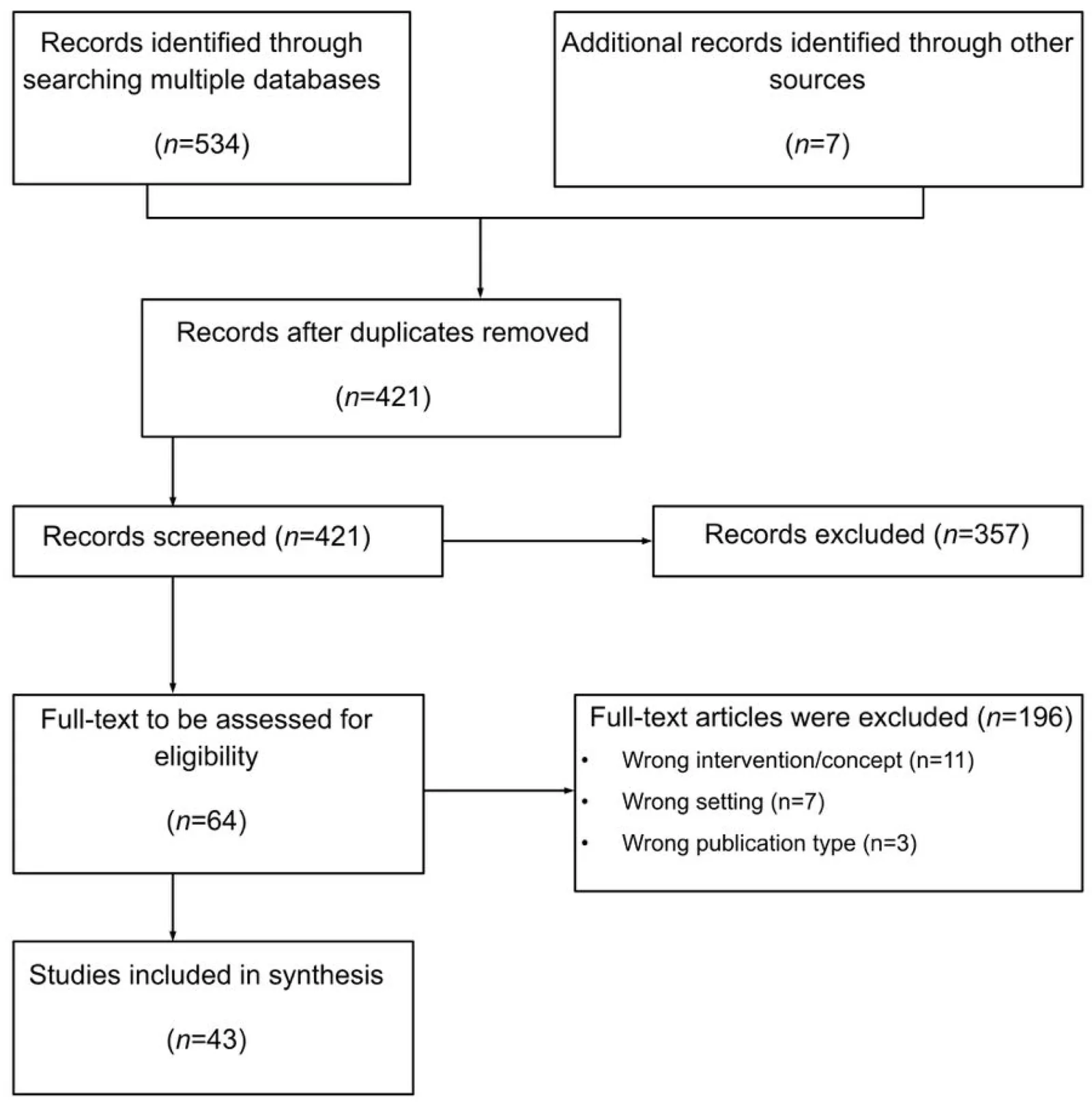
PRISMA flowchart depicting the search results.

The study selection process is illustrated in a flow diagram according to the PRISMA-ScR criteria [40] (Figure 1).

### 3.2. Evidence Distribution

**Figure 2:**
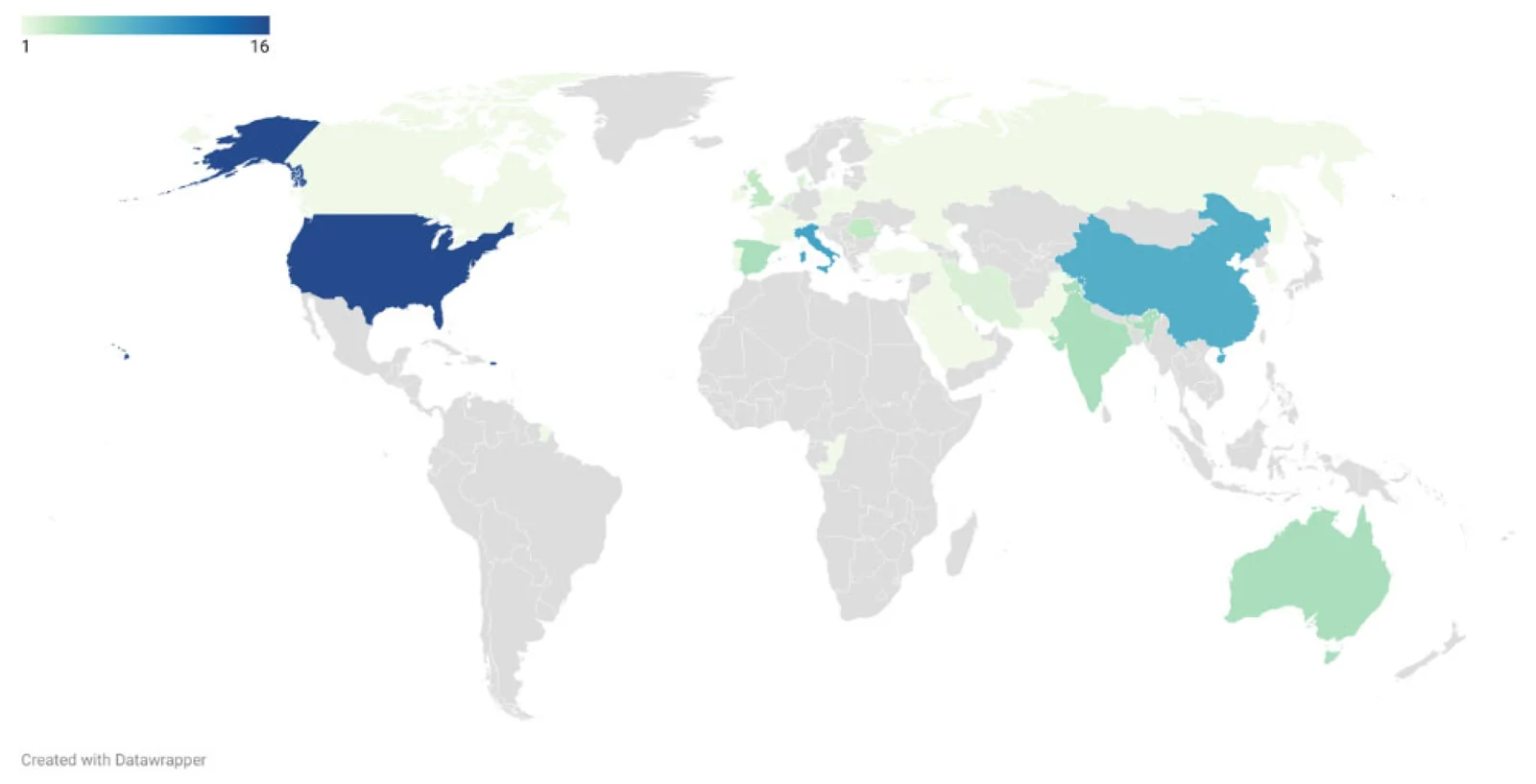
Distribution of selected studies by country.

The evidence presented encompasses a wide international distribution, covering 28 countries across five continents. Scientific production is predominantly led by the United States, Italy, and China, which concentrate the bulk of cutting-edge research on interventions such as Fecal Microbiota Transplantation (FMT), advanced metabolomics, and large-cohort longitudinal studies. Furthermore, a consistent contribution from various European and Asian centers was observed, along with a notable representation of studies from emerging economies. This geographical heterogeneity is of great methodological relevance, as it provides solid ecological validity to the global findings on the intricate interactions between dietary patterns, cultural environments, and microbiota modulation (Figure 2).

### 3.3. Methodological and Population Heterogeneity

Regarding the study design, the evidence was highly heterogeneous, following a pattern similar to that observed in reviews of complex chronic conditions. The body of evidence is primarily divided into the following categories:

Narrative and Systematic Reviews: These reviews synthesize the biological mechanisms underlying the gut–brain axis.

Observational and Cohort Studies: These link taxonomic composition (e.g., levels of Bacteroides or Akkermansia) with clinical progression as measured by the ALSFRS-R scale.

Clinical Trials and Interventions: There is a growing interest in Fecal Microbiota Transplantation (FMT) and modified diets (ketogenic or Mediterranean) to modulate affective states and neuroinflammation.

The study samples focused on adults with ALS, with specific attention given to the phenotypic differentiation between bulbar and spinal onset. This distinction is crucial because the clinical context (e.g., swallowing difficulties or gastric motility) directly affects the nutritional and psychological variables analyzed in this review (Table 2).

Rather than analyzing the findings geographically, the literature was synthesized thematically to prioritize biological relevance and mechanistic clarity across international cohorts. Studies have consistently demonstrated that gut dysbiosis in ALS is driven by core functional perturbations, regardless of geographic region. Consequently, the results are organized around five primary biological pillars: (1) taxonomic shifts in key microbial populations (e.g., *Firmicutes/Bacteroidetes* ratio and *Actinobacteria* depletion), (2) functional metabolic alterations (primarily short-chain fatty acids, tryptophan-kynurenine, and bile acid pathways), (3) mucosal barrier integrity and systemic endotoxemia (LPS translocation), (4) neuroinflammatory and immune signaling cascades (microglial M1 polarization, reactive astrogliosis, and cGAS-STING pathways), and (5) therapeutic implications and clinical translational barriers. This thematic integration provides a streamlined, pathophysiologically focused framework that overcomes population-specific descriptive biases.

### 3.4. Thematic Mapping Using the WHO ICF Framework

Current research on the microbiota–gut–brain axis in ALS necessitates the implementation of hybrid methodological protocols to reconcile scientific rigor and technical complexity of the hospital environment with ambulatory monitoring strategies. This approach results in a reduced burden for patients experiencing progressive deterioration of mobility [42].

The hospital environment centralizes the execution of invasive interventions, collection of systemic biological samples, and safety monitoring. Microbiome neuromodulation procedures, such as fecal microbiota transplantation (FMT) and washed microbiota transplantation (WMT), require specialized clinical infrastructure, as fecal preparations from healthy donors are administered via colonoscopy, endoscopy, or the use of transendoscopic enteral tubes [42,44,69]. Similarly, longitudinal clinical evaluation necessitates periodic in-person visits for the application of standardized functional scales (e.g., ALSFRS-R), respiratory function assessments (spirometry), and the extraction of serum or cerebrospinal fluid intended for the quantification of biomarkers, such as neurofilaments [42,45].

Conversely, to enhance adherence and obtain data that accurately reflect patients’ baseline conditions, a substantial portion of data collection and therapeutic interventions have transitioned to the home environment. In microbiomic procedures, patients are required to collect their own fecal samples at home using standardized kits [42,46]. To broaden the scope of epidemiological research, numerous studies have facilitated remote screening and enrollment of participants via online platforms and phone evaluations, effectively minimizing obstacles related to location and movement [42]. Finally, addressing hypermetabolism and dysbiosis through nutrition requires sustained execution in the patient’s daily life. Strategies such as adherence to a normocaloric ketogenic diet, Mediterranean diet, or intake of pre- and probiotic supplements have been implemented and evaluated in an ambulatory manner [43,45]. Patient adherence to these therapies is monitored at home using dietary records. Patients visit clinics or care networks solely for reassessment of metabolic and nutritional control.

The systematic analysis of the literature allowed for the categorization of symptomatology, pathophysiological alterations, and risk factors associated with ALS using the standardized framework of the WHO International Classification of Functioning, Disability, and Health (ICF) [70]. The results are divided into three major dimensions that articulate the complexity of the disease (Table 3): (1) Body Functions (b-codes), ranging from motor and respiratory deficits to neurocognitive and gut–brain axis alterations; (2) Body Structures (s-codes), focused on neurodegeneration and the integrity of the intestinal barrier; and (3) Environmental Factors (e-codes), which integrate both therapeutic nutritional support and the impact of the exposome on health.

#### 3.4.1. Body Functions: Multisystemic Involvement

At the neuromuscular level, patients present with profound impairment of muscle power functions (b730), characterized by progressive weakness and paralysis that compromise both the limbs and bulbar musculature [43,50,51,71]. This clinical presentation is accompanied by intrinsic alterations in muscle tone function (b735), evidenced by spasticity, rigidity, hypertonia, and fasciculations derived from the degenerative process [51,71,72]. The involvement of the bulbar segment directly impacts ingestion functions (b510), manifesting as severe dysphagia and sialorrhea [50,52,71], as well as articulation of speech functions (b320), leading to dysarthria [42,71]. Critically, for survival, there is a relentless deterioration of respiratory function (b440), leading to progressive respiratory failure marked by a decrease in forced vital capacity (FVC) [41,43,45,71].

Within the context of the gut–brain axis, a general deterioration of digestive functions and peristalsis (b515) was established, encompassing delayed gastric emptying, anomalies in bile acid metabolism, and a pathological increase in intestinal permeability or leaky gut [53,54,55]. This gastrointestinal dysfunction is closely related to alterations in defecation functions (b525), with reports of severe chronic constipation mediated by intestinal dysbiosis [41,42,55]. Simultaneously, a marked deficit is observed in weight maintenance functions (b530) due to an underlying hypermetabolic state, which promotes accelerated loss of body mass and clinical malnutrition, predicting a shorter survival [45,52,56].

In the neuropsychological sphere, impairment of higher-level cognitive functions (b164) was identified, manifesting as cognitive decline and behavioral alterations, with a demonstrated clinical and genetic overlap with Frontotemporal Dementia [46,57,73]. Additionally, there is a deterioration of emotional function (b152), characterized by the presence of emotional instability, anxiety, and clinical signs of depression that severely impact the quality of life [42,43,71].

#### 3.4.2. Body Structures

The morphological substrate of the disease is reflected in direct cellular damage, atrophy, and localized degeneration within the structure of the brain and spinal cord (s110/s120), specifically affecting the upper motor neurons of the cerebral cortex and lower motor neurons of the brainstem and spinal cord [43,50,51,71]. In parallel, evidence underscores severe extraneurological damage to the structure of the intestine (s540), characterized by damage to the intestinal mucosa, alteration of Paneth cells, destruction of tight junctions (zonulin), and involvement of the Enteric Nervous System [52,54,55].

#### 3.4.3. Environmental Factors

The pathogenesis and clinical course of ALS are strongly modulated by contextual factors. The findings highlight the key impact of products or substances for personal consumption (e110), evidencing the prognostic relevance of interventions focused on clinical nutrition (such as ketogenic and Mediterranean diets and pre/probiotics) and Fecal Microbiota Transplantation (FMT) [43,45,56]. Conversely, the analysis of the exposome reveals that determinants of the physical environment (e250/e260), such as historical patient exposure to heavy metals, pesticides, electromagnetic radiation, and specific environmental toxins, confer significant exogenous risk [51,58,68].

**Figure 3:**
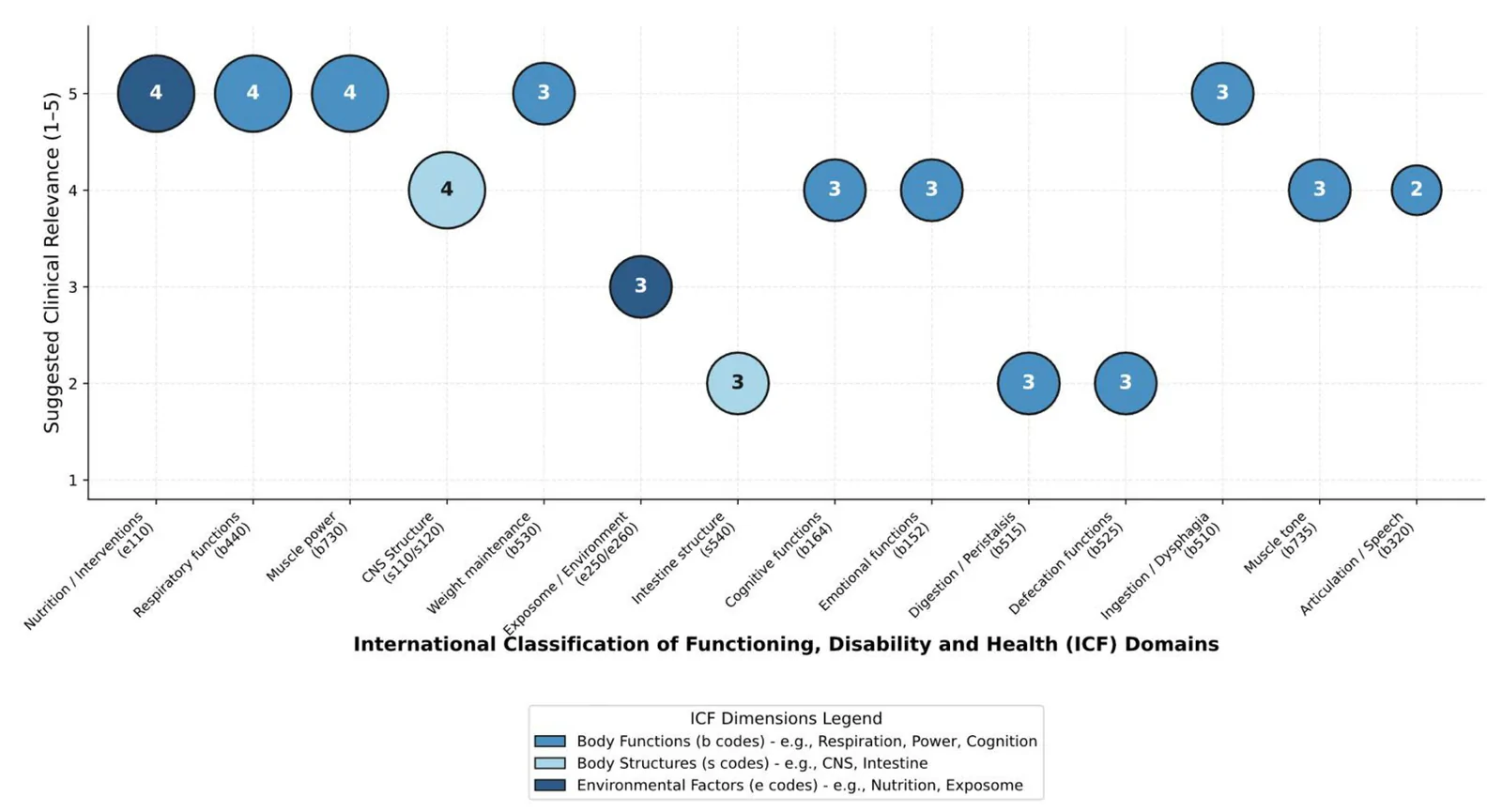
Densitometric mapping of Scientific Evidence in ALS according to ICF Domains. The size of each bubble is proportional to the number of independent studies identified in the systematic review that supported each domain; the number shown inside each bubble indicates this same count of supporting studies. The vertical position reflects the suggested clinical relevance of each domain (scale 1–5). The colors group the domains by functional systems. A saturation of evidence is observed in respiratory, motor, and nutritional functions, while critical domains, such as the articulation of speech (b320), present a significant knowledge gap.

Figure 3 illustrates the evidence mapping based on ICF domains. A methodological asymmetry is observed, where classic motor and respiratory functions predominate in the current literature. Conversely, a notable gap in knowledge exists in areas such as speech articulation (b320), alongside a requirement for a more detailed structural characterization of the intestine (s540) rather than solely its functional description. This analysis highlights the importance of incorporating comprehensive evaluations that consider both environmental factors, such as the exposome, and intestinal health as integral components in ALS research.

### 3.5. Facilitating Factors and Barriers in the Clinical and Methodological Approach to ALS (Table 4)

#### Facilitating Factors

Strategies oriented toward the modulation of the intestinal environment and optimization of research protocols have demonstrated significant positive impacts on patients with ALS, allowing for a more effective multidisciplinary approach [42,56]. At the clinical level, the implementation of therapeutic diets, such as the normocaloric ketogenic and Mediterranean diets, acts as a potent metabolic facilitator against underlying hypermetabolism [45,52]. On the one hand, the ketogenic diet provides motor neurons with alternative energy sources by inducing the biosynthesis of ketone bodies, such as 3-hydroxybutyrate, which compensates for the cellular energy crisis [45,74]. In contrast, dietary patterns rich in fiber, polyphenols, and healthy fats, typical of the Mediterranean diet, favorably modify the diversity of the microbial ecosystem [43,56]. Collectively, these dietary interventions substantially improve the integrity of the epithelial and blood-brain barriers, limiting damage caused by intestinal permeability (leaky gut), and drastically attenuating the neuroinflammation cascade [54,75].

Complementarily, the targeted administration of prebiotics, probiotics, and bioactive plant molecules or antioxidant compounds—particularly polyphenols such as resveratrol, curcumin, and epigallocatechin gallate (EGCG)—exerts a strong immunomodulatory and dysbiosis-correcting action [53,55,71,76].

Beyond acting as key metabolic signals, short-chain fatty acids (SCFAs), particularly butyrate, exert profound epigenetic and cellular regulatory functions in the gut–brain axis. Butyrate acts as a potent endogenous histone deacetylase (HDAC) inhibitor, modifying chromatin structure through increased histone acetylation status to epigenetically regulate anti-inflammatory and structural gene expression [77,78].

Mechanistically, butyrate restores and maintains intestinal tight junction integrity through both direct epigenetic and indirect metabolic pathways:

Direct Epigenetic Regulation: As an HDAC inhibitor, butyrate enhances histone acetylation at the promoter regions of tight junction proteins—specifically Claudin-1, Occludin, and Zonula Occludens-1 (ZO-1)—thereby transcriptionally upregulating their expression and strengthening intercellular epithelial sealings [77].

Indirect Mitochondrial Improvement: Butyrate serves as the primary energy substrate for colonocytes via mitochondrial β-oxidation. By feeding the tricarboxylic acid (TCA) cycle and oxidative phosphorylation, it restores cellular ATP levels, attenuates mitochondrial ROS production, and stabilizes the energetic requirement necessary for the structural maintenance and assembly of tight junction complexes [78].

These protective mechanisms have been explicitly validated in SOD1G93A ALS transgenic mouse models, where targeted butyrate supplementation significantly restored intestinal epithelial integrity, normalized dysbiotic microflora, reduced systemic inflammation, and delayed neuromuscular disease progression [77,78].

Likewise, FMT is emerging as a therapeutic intervention with significant potential in the comprehensive management of the disease. Data from clinical trials and systematic reviews indicate that this approach facilitates the restoration of microbial ecosystem diversity, promoting an increase in beneficial strains, such as Bifidobacterium [41]. Although its impact on long-term motor symptom progression requires further investigation, FMT results in significant clinical improvement of non-motor symptoms in ALS. Specifically, its efficacy has been demonstrated in alleviating gastrointestinal alterations such as chronic constipation and notably reducing markers of emotional distress, as assessed by anxiety and depression scales [41,56,60].

In the context of the environmental exposome, historical exposure to toxic xenobiotics constitutes a fundamental exogenous determinant in ALS pathogenesis [51,58]. Alongside heavy metals, agrochemicals, and smoking, specific environmental neurotoxins play a critical role [51,58]. Notably, gyromitrin and its toxic metabolite monomethylhydrazine (MMH)—naturally occurring hydrazines found in certain edible fungi like *Gyromitra esculenta*—have been identified as key environmental risk factors capable of inducing persistent metabolic dysfunction, oxidative stress, and neurotoxicity [79].

Furthermore, the neurotoxin β-methylamino-L-alanine (BMAA) provides a classic paradigm of environmentally driven neurodegeneration [53,58,70]. BMAA, synthesized by cyanobacteria and bioaccumulated in cycad seeds (*Cycas micronesica*), was historically consumed by the indigenous Chamorro population of Guam via traditional foodstuffs (e.g., cycad flour and fruit bats that feed on cycads) [80]. This bioaccumulation established a direct causative link to the extraordinarily high incidence of Amyotrophic Lateral Sclerosis/Parkinsonism-Dementia Complex (ALS/PDC) in Guamanians [79]. Mechanistically, BMAA misincorporates into host cellular proteins, triggers protein misfolding, interacts with gut microflora, and activates glutamate receptors to induce excitotoxicity and severe neuroinflammation [52,57,64].

Additionally, neurotoxins such as BMAA, produced by cyanobacteria and present in certain food chains, interact with the gut microbiota and can be anomalously incorporated into host cell polypeptide chains. This mechanism promotes protein misfolding, activates glutamate receptors—triggering excitotoxicity—and initiates a marked neuroinflammatory response [52,57,64].

This convergence of environmental alterations synergistically exacerbates accumulated neurotoxic risk, catalyzing glia-mediated inflammation and accelerating the irreversible process of motor neuron degeneration [54,56].

The presence of bulbar and gastrointestinal symptomatology constitutes one of the primary physiological barriers to the effective implementation of supportive therapies in ALS patients. Specifically, progressive severe dysphagia—frequently accompanied by anorexia, chewing impairments, and orofacial dysfunction (ICF domain: b510)—drastically compromises voluntary caloric intake, imposing mechanical and behavioral impediments that severely limit adherence to oral nutritional interventions [50,52,56].

Concurrently, this nutritional depletion aggravates the basal hypermetabolism characteristic of the disease (b530), triggering an underlying energy deficit cascade. This imbalance accelerates the depletion of lean body mass and body fat, precipitating a state of clinical malnutrition that is widely recognized as an independent risk factor and a negative prognostic predictor strongly associated with faster disease progression and lower overall survival [56,57,58].

At the tissue and immunological levels, these alterations converge with marked dysbiosis and structural deterioration of the gastrointestinal epithelium’s tight junctions, causing a pathological increase in mucosal permeability (leaky gut (b515)). This disruption of mucosal barrier integrity facilitates the systemic translocation of pathogenic microorganisms and bacterial toxins—particularly lipopolysaccharides—into the bloodstream [81].

This disruption of mucosal barrier integrity facilitates the systemic translocation of pathogen-associated molecular patterns—particularly lipopolysaccharides (LPS)-into the bloodstream [55,66]. Systemic LPS induces TLR4-mediated microglial hyperactivation, exacerbating neuroinflammation [55,66]. Notably, central nervous system vascular compromise and blood–brain barrier (BBB) degradation occur in parallel through distinct mechanisms: primarily driven by the loss and degradation of endothelial tight junction proteins (exacerbated by mutant protein aggregate accumulation) and the focal downregulation or mislocalization of Aquaporin-4 (AQP4) expression in astrocytic end-feet surrounding the brain microvasculature [81].

### 3.6. Characterization of Dysbiosis and Its Correlation with Neuropsychiatric Symptomatology

ALS is currently recognized as a multisystem disorder in which non-motor symptoms have a substantial impact; specifically, the prevalence of depression reaches approximately 44%, and anxiety 33% [41]. This neuropsychiatric symptomatology occurs in conjunction with marked intestinal dysbiosis, which exhibits a profile partially distinct from that of other neurodegenerative diseases [60].

In ALS, an alteration in the Firmicutes/Bacteroidetes ratio is observed, alongside a severe depletion of butyrate-producing taxa—such as *Eubacterium rectale* and *Roseburia* spp.—and a concomitant enrichment of pro-inflammatory bacterial families [60]. Furthermore, a high abundance of potentially pathogenic microorganisms, including Escherichia coli and various species of the Enterobacteriaceae family, has been established [53]. Conversely, the presence of certain beneficial bacteria correlates with improvements in emotional state; for instance, the abundance of Akkermansia muciniphila is linked to increases in the hippocampal GABA/glutamate ratio [53], while an increase in the Actinobacteria phylum is associated with improvements in inflammation models related to depression [82].

### 3.7. Pathogenic Pathways of the Microbiota–Gut–Brain Axis

The influence of the microbiome on the emotional distress of ALS patients is mediated by a complex bidirectional communication network involving neuroendocrine, immunological, and metabolic factors.

On one hand, responses to emotional stress impact the limbic system and activate the hypothalamic–pituitary–adrenal (HPA) axis, triggering the release of cortisol [73]. This process exacerbates the increase in intestinal permeability (leaky gut syndrome), allowing the translocation of lipopolysaccharides and other bioactive molecules into the systemic circulation [66,73].

In contrast, dysbiosis favors the production of pathogen-associated molecular patterns that stimulate the release of pro-inflammatory cytokines, such as IL-1β, IL-6, and TNF-α [60,73]. Systemic translocation of gut-derived endotoxins triggers a widespread inflammatory cascade, wherein reactive microglial cells and astrocytes within the central nervous system, alongside circulating monocytes and tissue-resident/infiltrating macrophages in the periphery, serve as the primary cellular sources secreting elevated levels of key pro-inflammatory cytokines, including IL-1β, IL-6, TNF-α, and IL-18. Upon crossing the blood–brain barrier, these mediators promote the polarization of microglia toward a neurotoxic M1 phenotype, feeding back into the neural circuits associated with anxiety and depression [60].

In conjunction with the aforementioned factors, the gut microbiota synthesizes metabolites critical for neurological homeostasis. The loss of Bifidobacterium and Lactobacillus strains compromises the production of gamma-aminobutyric acid (GABA) and the availability of serotonin and dopamine precursors, thereby promoting excitotoxicity and mood dysfunction [60,73].

Likewise, the deficiency of short-chain fatty acids (SCFAs), particularly butyrate, eliminates their protective effect as histone deacetylase (HDAC) inhibitors, which is essential for promoting the differentiation of regulatory T cells (Tregs) and fostering an anti-inflammatory M2 microglial state [60].

### 3.8. Therapeutic Modulation of Emotional Distress Through Microbiome Interventions

The management of emotional distress in ALS through the modification of the microbiota–gut–brain axis shows promising preclinical and clinical results. Randomized controlled trials have demonstrated that FMT induces significant and quantifiable improvements in psychiatric symptoms, with an observed decrease in Hamilton Depression Rating Scale and anxiety scale scores [41]. This psychological benefit correlates with sustained changes in the community microbial composition, most notably marked by an increase in the abundance of the genus Bifidobacterium, known for its “psychobiotic” potential to attenuate depression [41].

Furthermore, the use of bioactive compounds favorably modulates microbial ecology. The literature highlights the therapeutic potential of epigallocatechin gallate (EGCG), curcumin, and resveratrol [53]. In particular, the administration of EGCG significantly promotes the growth of Akkermansia muciniphila and the Verrucomicrobia phyla, inducing a reduction in systemic inflammatory cytokines and improving the neurobiological environment related to mood [53].

### 3.9. Impact of Biological Sex on Nutritional and Pharmacological Bioavailability During ALS Progression: Animal Models

Recent scientific evidence, derived from proteomic analysis of animal models, specifically SOD1G93A mutation mice, indicates that biological sex exerts a significant and highly specific influence on alterations in intestinal absorption during the progression of ALS [83]. Alterations in the molecular processes that control absorption and metabolism in the small intestine are primarily observed in males. While significant alterations in the small intestine proteome are scarcely detected in females—isolating only an increase in copper chaperone protein (CCS)—males with ALS exhibit significantly altered expression of multiple transporters and enzymes [83].

Specifically, a significant overexpression of the SLC5A1 and NPC1L1 transporters, which are responsible for dietary absorption of glucose and cholesterol, respectively, has been observed in males. Likewise, they exhibit an increase in the SLC5A4A and ANO6 transporters, which could directly alter the uptake of ions such as calcium, chloride, or sodium through the intestinal epithelium.

Regarding drug absorption and metabolism, males show a significant increase in the SLC15A1 transporter (also known as PEPT1)—a key pathway for the absorption of oral medications and peptides—and the overexpression of metabolizing enzymes such as CYP4B1 and GLUD1.

## 4. Discussion

This study aimed to identify and characterize the factors determining emotional distress in ALS via the MGB axis. This scoping review highlights that emotional distress and neuropsychiatric symptomatology (such as anxiety and depression) in the ALS population should not be addressed in isolation as a mere reactive epiphenomenon to progressive motor impairment. Instead, they possess a tangible biological and neurochemical substrate mediated, in part, by the alteration of the microbiota–gut–brain axis [18,41,53], which is in line with other studies [21,84,85]. M1 microglia cause motor neuron damage through the release of pro-inflammatory cytokines (TNF-α, IL-1β, IL-6), ROS/RNS production, and glutamate excitotoxicity. Furthermore, ferroptosis—an iron-dependent cell death driven by lipid peroxidation and GPX4 dysfunction—serves as a key trigger that sustains M1 microglial activation and accelerates motor neuron death [86].

Unlike other cohorts with neurodegenerative diseases (such as Alzheimer’s or Parkinson’s), the evidence demonstrates that ALS patients present a critical and distinctive dysbiotic phenotype [60]. Recent human studies consistently identify a distinct microbial pattern when compared to healthy controls and other neurological pathologies. This finding supports the hypothesis of a specific dysbiotic “signature” associated with the disease [20,42,87]. Beyond descriptive association, bidirectional Mendelian randomization analysis supports a causal contribution of specific microbial taxa and lipid mediators to ALS risk [20].

This dysbiosis is characterized by a significant alteration in the *Firmicutes/Bacteroidetes* ratio and a severe depletion of key short-chain fatty acid (SCFA)-producing strains, notably the pathological decrease in butyrate-producing taxa such as *Roseburia intestinalis* and *Eubacterium rectale* [42,64,84]. In addition to its role as an energetic substrate, butyrate functions as a critical histone deacetylase (HDAC) inhibitor that epigenetically regulates gut barrier integrity and immune responses [77,78]. Specifically, butyrate directly upregulates the expression of tight junction proteins (Claudin-1, Occludin, and ZO-1) via increased histone acetylation at their promoter regions, while indirectly preserving epithelial integrity by fueling colonocyte mitochondrial β-oxidation, reducing ROS, and mitigating mitochondrial stress [78]. Studies in SOD1G93A transgenic ALS mouse models demonstrate that restoring butyrate levels reverses intestinal leakiness, dampens systemic inflammation, and delays neurodegeneration [47,77,88]. Consequently, the depletion of butyrate-producing taxa in ALS patients directly compromises these dual epigenetic and bioenergetic protective shields, aggravating both mucosal damage and systemic endotoxemia.

At a pathogenic level, this microbial ecosystem dysfunction interacts synergistically with the underlying hypermetabolism state characteristic of the disease. This convergence fosters a systemic energy deficit which, combined with the deprivation of neuroprotective metabolites and intestinal permeability, not only accelerates the patient’s clinical and motor decline but also feeds back into systemic neuroinflammation and profoundly exacerbates neuropsychiatric symptomatology [56,57].

Recent reviews on the brain–gut–microbiota axis in Amyotrophic Lateral Sclerosis (ALS) and affective disorders have elucidated the mechanisms by which dysbiosis, increased intestinal permeability, and translocation of lipopolysaccharides contribute to chronic low-grade neuroinflammation. This inflammation is linked to the progression of these diseases and the emergence of depressive and anxiety symptoms [12,89].

Regarding the core concept of the study, the evidence consolidates that the disruption of the microbiota–gut–brain axis acts as a primary catalyst for systemic neuroinflammation, which underlies the etiology of the neuropsychiatric symptomatology frequently observed in ALS [63,65]. The dysbiotic environment promotes a pathological increase in epithelial barrier permeability, thereby facilitating the systemic translocation of lipopolysaccharides (LPS) and other bacterial endotoxins into the bloodstream [43,54,66]. Crucially, recent mechanistic insights indicate that downstream of TLR4-mediated endotoxemia, cytosolic sensing of translocated bacterial factors and damaged cellular DNA activates the cGAS-STING signaling pathway within central microglia and peripheral monocytes [88]. This pathway serves as an innate immune sensor that orchestrates microglial and macrophage polarization toward neurotoxic M1 phenotypes while suppressing protective M2 states. The activation of cGAS-STING generates a persistent feed-forward inflammatory loop that continuously amplifies central neurodegeneration and feeds back into the neural circuits governing emotional distress and mood dysregulation [66,73,80]. The results align with the research conducted by Chen et al., Cheng et al. and Evrensel et al. [12,90,91].

Furthermore, while pro-inflammatory cytokines such as IL-1β, TNF-α, and IL-6 are established mediators in ALS pathogenesis, the mechanistic complexity of cytokine signaling—particularly regarding IL-6—warrants deeper articulation [92]. IL-6 exhibits a dual, context-dependent nature: classically acting as an exercise-induced myokine that promotes metabolic homeostasis and physiological muscle adaptation through anti-inflammatory classical membrane-bound signaling, versus functioning as a pathological driver of neuroinflammation and muscle atrophy via soluble receptor-mediated trans-signaling [92]. This distinction helps reconcile paradoxical cytokine findings across the ALS literature, highlighting how chronic systemic trans-signaling shifts IL-6 from a metabolic facilitator to a mediator of catabolic tissue wasting. Moreover, this inflammatory cascade engages in critical crosstalk with endoplasmic reticulum (ER) stress pathways within motor neurons and glial cells. Peripheral endotoxemia and persistent trans-signaling exacerbate unfolded protein responses (UPR), creating a metabolic and proteostatic strain that directly accelerates motor neuron degeneration [93].

To achieve a truly holistic and stratified therapeutic approach, it is essential to integrate the patient’s historical exposome [54,58]. Accumulated exposure to heavy metals, pesticides, or specific environmental toxins—such as fungal gyromitrin/MMH and bioaccumulated cyanobacterial BMAA from cycad seeds (which historically drove the ALS/PDC endemic in Guam)—exacerbates neurotoxic risk, compromises mucosal barrier integrity, and induces long-term intestinal dysbiosis [79]. These environmental factors introduce critical statistical noise and biological heterogeneity that must be strictly monitored in clinical trials [54,58].

Our results are consistent with those of recent studies that have identified a link between the prevalence of certain bacterial strains, particularly Akkermansia muciniphila, and an increase in the hippocampal GABA/glutamate ratio [47,48,94,95]. This suggests a psychobiotic mechanism that may reduce excitotoxicity and emotional disturbance [50,53].

From this perspective, the integration of the International Classification of Functioning, Disability and Health (ICF) framework is revealing: while the impact of the disease on muscle power functions (domain b730) has traditionally dominated the scientific literature [51,71], the prognostic correlation between the loss of integrity of the intestinal structure (domain s540) and the development of emotional distress constitutes a pressing knowledge gap that demands further clinical and translational exploration [41,52,55].

From an integrative clinical and ecological perspective, current evidence justifies the need to incorporate microbiome modulation into clinical practice guidelines and advanced ALS care, both in hospital and home settings [56]. The preliminary efficacy of emerging interventions, such as FMT and the prescription of therapeutic diets (ketogenic and Mediterranean), has revealed a promising dual impact.

These strategies modulate the course of neurodegeneration by providing alternative energy substrates to counter the hypermetabolic deficit [45]. On the other hand, they achieve an objective impact on neuropsychiatric and emotional involvement; while FMT intervention has been shown to significantly attenuate anxiety and depression as assessed by standardized scales [41]. Interventions based exclusively on the Mediterranean diet have succeeded in maintaining stable symptomatology, with a slight downward trend according to the Beck Depression Inventory [43].

Nevertheless, the provision of these cares faces complex pathophysiological barriers intrinsic to ALS. The progression of severe dysphagia (ICF domain: b510) and the development of anorexia drastically complicate the viability of and adherence to any nutritional or symbiotic therapy administered orally [54,56]. This functional limitation highlights the urgent need to adopt adapted and innovative administration protocols.

The use of transendoscopic enteral tubes emerges as a highly effective logistic and therapeutic mechanism to bypass bulbar impairment, ensuring the safe and sustained delivery of both nutritional support and therapeutic fecal infusions directly into the intestinal tract [22,69].

Finally, to achieve a truly holistic, stratified, and personalized therapeutic approach, it is essential to integrate the analysis of the patient’s historical exposome. Accumulated exposure to environmental toxins, heavy metals, or previous antibiotic treatments not only exacerbates neurotoxic risk but also fundamentally alters the intestinal ecosystem, introducing considerable statistical noise and confounding factors that must be strictly monitored in the design of future clinical trials [54,58].

In this regard, various reviews have highlighted that both exposure to environmental pollutants and heavy metals, as well as the repeated use of antibiotics, can induce persistent dysbiosis, modify microbiota composition, and be associated with neurocognitive and affective alterations, reinforcing the need to consider the exposome as a key modulator of the microbiota–gut–brain axis [49,96,97].

Accumulating evidence shows that pathognomonic protein aggregation in ALS extends beyond the central nervous system, supporting a peripheral proteinopathy model. Notably, phosphorylated TDP-43 (pTDP-43) deposits in peripheral tissues, such as cutaneous nerve fibers [98], link systemic immune dysregulation to central neurodegeneration. Peripheral pTDP-43 pathology triggers local inflammation, impairs axonal transport, and interacts with gut-derived endotoxins (LPS), synergistically accelerating motor neuron loss. In addition to changes in intestinal transporters [83], biological sex plays a crucial role in influencing the onset and progression of ALS. Sex-dependent immunometabolic mechanisms, including macrophage polarization, metabolic reprogramming [99], and catabolic muscle atrophy pathways [100], dictate neurodegenerative susceptibility, highlighting sex stratification as essential for precision ALS trials.

### 4.1. Mechanistic Convergence of Therapeutic Interventions: Energy Sensing, Metabolic Reprogramming, and Macrophage Polarization

While clinical and preclinical evidence supports the utility of dietary (ketogenic and Mediterranean diets) and microbiome-based interventions (FMT and psychobiotics) in ALS, articulating their underlying downstream signaling cascades is essential to establish a robust translational rationale.

A central molecular axis where these metabolic and nutritional interventions converge is the AMPK/SIRT1/PGC-1α pathway. AMPK serves as a pivotal cellular energy sensor that, upon activation by energy stress or specific dietary regimens such as the ketogenic diet, upregulates SIRT1 and PGC-1α. In the context of ALS pathophysiology, where motor neurons and peripheral tissues suffer from severe hypermetabolism, mitochondrial dysfunction, and oxidative stress, the activation of the AMPK/SIRT1/PGC-1α axis acts as a metabolic master regulator. It promotes mitochondrial biogenesis, restores cellular ATP homeostasis, and suppresses nuclear factor kappa B (NF-κB)-mediated pro-inflammatory cascades [101] Thus, ketogenic diets and SCFA-producing interventions (e.g., butyrate restoration via Mediterranean diet or FMT) do not merely supply alternative metabolic substrates like ketone bodies; they actively engage this pathway to attenuate neuroinflammation and enhance neuronal bioenergetics.

Concurrently, modulating systemic and tissue-resident immune cells represents a primary therapeutic target of gut–brain axis interventions. Macrophages and microglia demonstrate significant plasticity, enabling them to transition between pro-inflammatory (M1) and tissue-reparative or anti-inflammatory (M2) phenotypes. As highlighted by Shang et al. [102], macrophages play a dual and context-dependent role in skeletal muscle atrophy and neuromuscular degeneration. Persistent M1 polarization driven by gut-derived endotoxemia (LPS/TLR4) exacerbates muscle catabolism and motor neuron damage. Therapeutic modulation through psychobiotics, FMT, or metabolic activation of the AMPK pathway shifts macrophage polarization from an M1 catabolic state to an M2 neuroprotective profile. This phenotypic switch mitigates inflammatory muscle wasting, promotes tissue regeneration, and halts the feed-forward inflammatory loops driving central neurodegeneration.

However, despite the compelling mechanistic rationale provided by preclinical models, the clinical translational evidence supporting FMT, psychobiotics, ketogenic regimens, or Mediterranean dietary patterns in ALS remains strictly preliminary. Currently, randomized, double-blind, placebo-controlled clinical trials involving human cohorts are limited. These trials are constrained by small sample sizes and have not yet established definitive disease-modifying efficacy or long-term clinical survival benefits for these drugs. Consequently, these approaches must be evaluated as complementary, exploratory strategies rather than validated disease-modifying therapies, avoiding unrealistic expectations for clinical practice until robust phase III trials are completed.

### 4.2. Oxidative Stress as a Central Pathophysiological Bridge: Gut Endotoxemia, Nrf2 Signaling, and Muscle-Neuronal Degeneration

Although oxidative stress is a well-recognized feature of ALS, its role as a pivotal converging mechanism linking intestinal dysbiosis to central and peripheral degeneration requires explicit articulation. The breakdown of the intestinal epithelial barrier and subsequent translocation of gut-derived endotoxins (e.g., LPS) into the systemic circulation trigger a pro-inflammatory state that drastically increases the generation of reactive oxygen species (ROS). As reviewed by Zhang et al. [78], oxidative stress is a critical driver of skeletal muscle atrophy and degeneration, establishing pernicious crosstalk with mitochondrial dysfunction, persistent inflammation, and activation of proteolytic systems (such as the ubiquitin-proteasome system and autophagy-lysosome pathways). In ALS, this systemic oxidative burden exacerbates both motor neuron cell death and non-motor clinical manifestations.

Crucially, the gut microbiota plays a major regulatory role in modulating host antioxidant defenses, primarily through the nuclear factor erythroid 2-related factor 2 (Nrf2) pathway. Beneficial microbiota-derived metabolites, particularly short-chain fatty acids (SCFAs like butyrate) and dietary polyphenols transformed by gut microbes, serve as key activators of Nrf2 signaling. Upon activation, Nrf2 translocates to the nucleus and drives the transcription of essential phase II antioxidant enzymes (e.g., HO-1, NQO1, SOD1), thereby neutralizing ROS, protecting mitochondrial integrity, and dampening NF-κB-driven neuroinflammation. Conversely, in the severe dysbiotic state characteristic of ALS—where SCFA-producing taxa are depleted and LPS translocation is heightened—this Nrf2-mediated antioxidant protection is severely compromised. This imbalance amplifies neuroinflammation, fuels microglial hyperactivation, and accelerates both muscle wasting and motor neuron loss, highlighting Nrf2 activation as a primary mechanistic target for gut–brain axis interventions.

It is critical to distinguish peripheral TLR4-mediated microglial activation from the structural and functional breakdown of the blood–brain barrier (BBB). While gut-derived LPS fuels microglial pro-inflammatory phenotypes, primary BBB breakdown is mediated by endothelial tight junction disassembly and the loss of polarized Aquaporin-4 (AQP4) water channels on astrocytic perivascular end-feet. This vascular disruption acts synergistically with reactive astrogliosis, a critical driver of non-cell autonomous motor neuron degeneration in ALS. Reactive astrocytes undergo a toxic loss of normal homeostatic functions—such as impaired extracellular glutamate clearance via downregulated EAAT2/GLT-1 transporters—leading to localized excitotoxicity. Concurrently, reactive astrocytes acquire non-cell autonomous neurotoxic properties, secreting pro-inflammatory cytokines, reactive oxygen species, and toxic factors that directly compromise mitochondrial function and induce apoptosis in upper and lower motor neurons [81]. Integrating these astrocytic and vascular mechanisms provides a more precise framework connecting peripheral endotoxemia with central neurodegenerative cascades.

### 4.3. Mechanistic Foundations of Psychological Manifestations: Neuroinflammation, Neurotransmitters, and Emotional Distress

Although emotional distress, anxiety, and depression in ALS patients are often viewed as secondary psychosocial reactions to progressive physical disability, emerging evidence indicates they are heavily driven by organic neuroinflammatory cascades along the gut–brain axis. Dysbiosis-induced systemic endotoxemia (LPS translocation) and central microglial activation upregulate pro-inflammatory cytokines (TNF-α, IL-1β, IL-6), which directly alter tryptophan metabolism by shifting it away from serotonin synthesis toward the neurotoxic kynurenine pathway. Furthermore, altered SCFA production impairs GABAergic and glutamatergic signaling within limbic circuits, disrupting emotional regulation. This biological crosstalk creates a self-propagating neuroinflammatory loop that exacerbates psychological vulnerability alongside motor decline, highlighting that neuropsychiatric symptoms in ALS represent an intrinsic neurobiological component of gut–brain axis disruption rather than purely reactive distress.

### 4.4. Clinical Translation: Biomarkers, Prognostic Value, and Barriers

To bridge the gap between descriptive dysbiosis and clinical practice, the translational potential of gut microbiota-derived signatures in ALS must be critically evaluated. Across independent international cohorts, the most consistent and reproducible microbial alterations involve a reduction in the *Firmicutes/Bacteroidetes* ratio, the depletion of short-chain fatty acid (SCFA)- and butyrate-producing taxa (specifically *Roseburia*, *Eubacterium*, and *Faecalibacterium*), and elevated systemic lipopolysaccharide (LPS) levels. Although these microbial signatures currently lack the diagnostic specificity required for standalone diagnosis due to overlap with other neurodegenerative disorders, they possess significant prognostic value, as baseline SCFA depletion and systemic endotoxemia directly correlate with accelerated ALSFRS-R functional decline and shorter patient survival. However, routine clinical implementation remains hampered by key barriers, including the lack of standardized sequencing and bioinformatic protocols, high inter-individual variability driven by diet, geographic location, and medication use, as well as strict regulatory and safety concerns regarding fecal microbiota transplantation (FMT) and live biotherapeutics in fragile ALS cohorts.

### 4.5. Critical Appraisal: Methodological Heterogeneity, Confounders, and Study Limitations

A critical synthesis of the ALS gut–brain axis literature highlights major methodological limitations and sources of bias that restrict comparability across cohorts. Technical discrepancies—such as 16S rRNA (V3–V4 vs. V4 regions) versus shotgun metagenomics, OTU vs. ASV resolution, reference database selection, and non-standardized stool handling—drive significant classification bias and account for contradictory findings regarding alpha diversity (Shannon/Chao1) and specific taxa (*Bacteroidetes*, *Akkermansia muciniphila*, *Ruminococcus*). Furthermore, unmonitored host confounders (diet, geographic location, BMI) and medications (riluzole, edaravone, antibiotics, laxatives) obscure true biological signals. Crucially, secondary disease manifestations—including dysphagia, altered gut motility, and enteral tube feeding (PEG)—introduce severe secondary bias. Because current evidence relies heavily on cross-sectional human cohorts and preclinical models (e.g., SOD1G93A mice), available data establish association rather than a proven causal relationship. Overcoming these constraints requires standardized protocols and longitudinal prospective trials to isolate primary drivers from secondary disease consequences.

Advancing the field requires four critical priorities: (1) longitudinal prospective cohorts to track early disease stages and establish temporal causality; (2) standardized methodologies for stool sampling, shotgun metagenomic sequencing, and ASV bioinformatic pipelines to eliminate technical inter-study noise; (3) multi-omics integration combining metagenomics with systemic/fecal metabolomics (SCFAs, tryptophan, bile acids) to map functional mechanisms; and (4) randomized, double-blind, placebo-controlled trials to rigorously evaluate whether gut-targeted interventions (probiotics, FMT, SCFA supplementation) hold true disease-modifying efficacy in ALS.

## 5. Conclusions

Accumulating research highlights a complex biological interplay between gut dysbiosis, systemic inflammation, and neurodegenerative pathways in ALS. However, current clinical evidence supports association rather than causation. While altered microbial profiles, loss of short-chain fatty acid-producing taxa, and elevated markers of gut permeability consistently correlate with disease progression, present data cannot establish whether gut dysbiosis acts as a primary etiology or as a secondary consequence of ALS progression, dysphagia, dietary shifts, and altered bowel motility. Therapeutic strategies targeting the gut–brain axis—such as dietary interventions, probiotics, or fecal microbiota transplantation—remain largely exploratory and lack definitive disease-modifying efficacy in human trials. Future research must move beyond cross-sectional profiling toward well-controlled longitudinal prospective cohorts and mechanistic interventional trials to determine whether modulating the gut microbiome can genuinely alter the clinical trajectory of ALS.

## Figures and Tables

**Table 1:** Breakdown of the research question according to the PCC framework.

Population (P)	Concept (C)	Context (C)
Adult patients with Amyotrophic Lateral Sclerosis (ALS)	Interaction between the gut microbiota (composition and function) and emotional distress (anxiety and depression).	Clinical and home settings, including nutritional factors and oxidative stress.

**Table 2:** Summary of clinical and preclinical studies and reviews on the impact of gut microbiota and nutritional interventions on ALS.

Intervention Type/Design	References	Estimated Population/Sample Size	Key Characteristics	Main Findings at the Group Level
Clinical Trial (FMT)	[41]	27 ALS patients (14 FMT, 13 placebo).	Fecal Microbiota Transplantation (FMT) from a healthy donor versus placebo (6 months).	FMT did not improve motor ALSFRS-R scores but significantly improved non-motor symptoms (constipation, anxiety, and depression) and increased *Bifidobacterium* levels.
Clinical Trial (Probiotics)	[42]	100 participants (50 ALS, 50 controls).	Longitudinal probiotic supplementation.	Positive modulation of the microbiota and intergroup changes influence the progression of impairment.
Longitudinal Study (Diet)	[43]	84 participants (44 ALS, 40 controls).	6-month Mediterranean Diet intervention and measurement of short-chain fatty acids (SCFAs).	The diet reduced plasma acetic and propanoic acid levels, and acetic acid levels were correlated with depression and fine motor dysfunction.
Clinical Protocol (FMT)	[44]	42 planned patients.	FETR-ALS trial involving healthy fecal microbiota infusion versus placebo.	Protocol: This study aimed to evaluate whether ketosis reduces neuroinflammation without causing weight loss and malnutrition.
Clinical Protocol (Diet)	[45]	25 planned ALS patients.	Normocaloric Ketogenic Diet for 8 weeks.	Protocol: This study aimed to evaluate whether ketosis reduces neuroinflammation without causing weight loss and malnutrition.
Observational/Microbiome	[11]	40 participants (10 ALS, 10 spouses, 20 healthy).	Fecal analysis controlling for environment/diet using partners as controls.	Higher alpha diversity in ALS patients, but with a marked deficiency of protective *Prevotella* spp.
Observational/Multi-omics	[20]	185 participants (75 ALS, 110 controls).	16S sequencing and interaction with the plasma lipid metabolome.	Decrease in butyrate-producing strains (*Lachnospiraceae*) and strong correlation between bacteria and plasma lipid/acylcarnitine profiles.
Observational/Metagenomics	[42]	139 participants (66 ALS, 61 healthy, 12 other pathologies).	Deep shotgun sequencing, evaluation of clinical variables, and constipation.	Lower presence of butyrate-generating strains (*Eubacterium rectale, Roseburia*) correlated with chronic constipation.
Observational/Microbiome	[46]	28 participants (16 recent ALS, 12 controls).	Recent symptom onset (<6–15 months), differentiating between bulbar and spinal phenotypes.	Early dysbiosis with increased *Fusobacteria*: microbiological changes restricted according to the ALS onset subtype.
Observational/Metabolomics	[47]	40 participants (20 ALS, 20 controls).	16S rRNA and stool metabolite chromatography.	Decreased Firmicutes/Bacteroidetes ratio and intracellular metabolic alterations.
Mendelian Randomization	[48]	Large-scale genetic GWAS data (thousands of patients).	Bidirectional analysis of microbiota and lipid metabolomics.	Causal confirmation: Microbiome alterations modify ALS risk using certain lipids as direct mediators.
Preclinical (Probiotic)	[49]	*C. elegans* nematode model (ALS).	Exposure to *Enterococcus faecium.*	Strong neuroprotection through the activation of the NHR-86 gene, mitigating reactive oxygen species (ROS) stress.
Reviews	[50,51,52,53,54,55,56,57,58,59,60,61,62,63,64,65,66,67,68]	Bibliographic consolidation and meta-analysis of thousands of human, in vivo, and in vitro studies.	Massive analysis of ALS pathogenesis linked to the gut microbiome and integrative therapies. Focus on barrier dysfunction (leaky gut), neuro-systemic inflammation (mediated by LPS and cross-translocation), gut–brain axis modulation, and the direct impact of ALS hypermetabolism. Studies the effects of FMT, diets (ketogenic, Mediterranean, hypercaloric), SCFAs (butyrate), pro/prebiotics, and vitamins on patient prognosis and progression.	Reviewed literature consistently reports profound ALS-associated dysbiosis: loss of butyrate-producing, neuroprotective taxa (*Akkermansia*, *Prevotella*, *Roseburia*) and increased pro-inflammatory/toxin-producing strains. Resulting barrier damage (‘leaky gut’) promotes LPS translocation, neuroinflammation, and motor neuron loss. Early combined nutritional (antioxidants, polyphenols, hypercaloric diet) and microbiota-restoring interventions (probiotics, FMT) emerge as a promising complementary strategy

**Table 3:** Distribution of studies according to the WHO ICF codes.

ICF Code	ICF Domain/Concept	Description of Involvement in ALS Articles	References
b730	Muscle power functions	Progressive loss of strength, muscle weakness, and paralysis affecting the limbs and bulbar muscles.	[50,51,71]
b735	Muscle tone functions	Emergence of spasticity, rigidity, hypertonia, and muscle fasciculations characteristic of neuronal degeneration.	[50,71,72]
b440	Respiratory functions	Progressive respiratory failure and critical decrease in forced vital capacity (FVC), which constitutes the main cause of mortality.	[42,71]
b510	Ingestion functions	Presence of dysphagia (severe difficulty swallowing) and sialorrhea (drooling) associated with the involvement of bulbar muscles.	[50,71,72]
b525	Defecation functions	Severe chronic constipation (assessed with scales such as Wexner or CSS), often related to intestinal dysbiosis.	[41,42,55]
b515	Digestive functions and peristalsis	General alteration of gastrointestinal function, including delayed gastric emptying, altered bile acid metabolism, and increased intestinal permeability (leaky gut).	[53,54,55]
b530	Weight maintenance functions	Underlying hypermetabolic state, accelerated loss of body mass, and clinical malnutrition that predicts shorter survival.	[54,55]
b152	Emotional functions	Presence of emotional instability, clinical signs of depression (Beck or HAMD scales), and anxiety that severely impact quality of life.	[41,71]
b164	Higher-level cognitive functions	Cognitive impairment and behavioral alterations, with a demonstrated clinical and genetic overlap with Frontotemporal Dementia (FTD).	[46,57,73]
b320	Articulation of speech functions	Dysarthria or severe speech problems caused by the bulbar onset of the disease.	[42,71]
s110/s120	Structure of the brain and spinal cord	Direct cellular damage, atrophy, and degeneration of upper motor neurons (cerebral cortex) and lower motor neurons (spinal cord and brainstem).	[50,51,71]
s540	Structure of the intestine	Damage at the level of the intestinal mucosa, alteration of Paneth cells, destruction of tight junctions (zonulin), and involvement of the Enteric Nervous System.	[52,54,55]
e110	Products or substances for personal consumption	Key impact of nutrition and therapeutic dietary interventions (e.g., ketogenic diet, Mediterranean diet, pre/probiotics, FMT).	[43,45,54,56]
e250/e260	Physical environment and Exposome	Exogenous risk conferred by historical patient exposure to heavy metals, environmental toxins (e.g., BMAA), pesticides, and electromagnetic radiation (exposome concept)	[51,58,68]

**Table 4:** Summary of facilitating factors and barriers in the clinical management of ALS.

Type	Category (ICF Domain)	Specific Factor	Description and Impact
Facilitator	Nutritional and Dietary (e110)	Therapeutic diets (Ketogenic, Mediterranean)	Alternative energy substrates (ketone bodies); improve gut barrier integrity and reduce systemic inflammation.
		Probiotics, prebiotics, and antioxidants (Polyphenols)	Metabolites such as butyrate restore tight junctions and confer neuroprotection
	Clinical and Therapeutic (e110)	Fecal Microbiota Transplantation (FMT)	Restores microbiome diversity; improves non-motor symptoms (constipation, depression, anxiety)
	Research and Methodology	Home-based adaptations	At-home sampling kits and remote recruitment enable participation despite progressive mobility loss.
Barrier	Environmental and Exposome (e250/e260)	Toxins, pollutants, and smoking, heavy metals, gyromitrin	Historical exposure (heavy metals, pesticides, BMAA) raises oxidative stress and neurotoxic risk.
	Physiological and Clinical (b510)	Dysphagia and loss of appetite	Impede oral intake and adherence to nutritional therapies.
	Physiological and Clinical (b530)	Hypermetabolism	Accelerates malnutrition and lean-mass loss; predicts shorter survival.
	Physiological and Clinical (b515)	Dysbiosis and leaky gut	Permits LPS/toxin translocation, feeding back into neuroinflammation.
	Research and Regulation	Lack of standardization and FMT biosafety	Absence of uniform protocols and infection risk limit clinical adoption.
	Methodological	Confounding factors in clinical studies	Genetics, antibiotic history and heterogeneous diets hinder causal inference in small samples.

## Data Availability

No new data were created or analyzed in this study. Data sharing is not applicable to this article.

## Supplementary Materials

The following supporting information can be downloaded at: https://www.mdpi.com/article/10.3390/pathophysiology33030059/s1, Supplementary File S1: Prisma checklist; Table S1: Results of the main studies and assessment of the risk of bias.

## Abbreviations

The following abbreviations are used in this manuscript:

AD	Alzheimer’s Disease
ALS	Amyotrophic Lateral Sclerosis
ALSFRS-R	Amyotrophic Lateral Sclerosis Functional Rating Scale-Revised
BMAA	β-Methylamino-L-alanine
CNS	Central Nervous System
CSF	Cerebrospinal Fluid
FMT	Fecal Microbiota Transplantation
FTD	Frontotemporal Dementia
FVC	Forced Vital Capacity
GABA	Gamma-aminobutyric acid
GI	Gastrointestinal
GWAS	Genome-Wide Association Study
ICF	International Classification of Functioning, Disability and Health
IL-6	Interleukin-6
LPS	Lipopolysaccharide
MGB	Microbiota–Gut–Brain axis
MR	Mendelian Randomization
PD	Parkinson’s Disease
ROS	Reactive Oxygen Species
SCFA	Short-Chain Fatty Acids
SOD1	Superoxide Dismutase 1
TNF-α	Tumor Necrosis Factor-alpha
WMT	Washed Microbiota Transplantation
WT	Wild-Type
