# Supplementary material for: The Microbiota–Gut–Brain Axis and Nutritional Interventions in Amyotrophic Lateral Sclerosis: Pathophysiological Mechanisms, Neuroinflammation, and Non-Motor Manifestations—Scoping Review
Source: Pathophysiology. 2026 Aug 10;33(3):59. doi: 10.3390/pathophysiology33030059 (PMC13511448; doi:10.3390/pathophysiology33030059)
Supplement: Supplementary file 1 [file pathophysiology-33-00059-s001.zip › pathophysiology-4423840-Table S1.pdf]

Table S1. Results of the main studies and assessment of the risk of bias.

Reference	Study Design	Sample Size (N)	Clinical Characteristics (ALS)	Microbiota Assessment	Nutritional Variables	Psychological Variables	Microbiota Findings	Clinical Correlations	Conclusions	JBI Bias Risk
**[67]**	Review (ALSUntangled data)	N/A (Review)	ALS (review of alternative / off-label therapies)	N/A	Off-label supplements and drugs (L-serine, methylcobalamin, etc.)	N/A	Several off-label treatments (e.g., L-serine, carnitines, antiretrovirals) have a documented capacity to alter and modulate the composition of the gut microbiome.	N/A	8 off-label treatments act on overlapping pathological mechanisms; combined trials suggested	Low
**[70]**	Review	N/A (Review)	ALS (human and SOD1G93A mouse models).	Review of previous methods (16S rRNA sequencing, metagenomics).	Prebiotics, probiotics, and postbiotics (e.g., short-chain fatty acids such as butyrate).	N/A	A decrease in the Firmicutes/Bacteroidetes ratio and in butyrate-producing bacteria (Anaerostipes, Oscillobacter) is reported. Positive modulation observed after supplementation with Akkermansia muciniphila.	The increase in intestinal permeability (leaky gut) promotes systemic inflammation that affects the blood-brain barrier. Butyrate correlates with an increase in regulatory T cells.	Immunonutritional interventions (pro-, pre-, and postbiotics) offer promising and innovative therapeutic options to slow neurodegeneration in ALS by modulating the gut-brain axis.	High

**[74]**	Narrative Review	N/A (Review)	ALS (humans and in vivo/in vitro models).	Review of the impact of bioactive compounds on the microbiota	Polyphenols (Flavonoids: quercetin, EGCG, naringin, hesperidin; non-flavonoids: gallic acid, curcumin, resveratrol, pterostilbene).	N/A	ALS is characterized by low levels of Butyrivibrio fibrisolvens and an increase in pathogens such as E. coli or Ruminococcus torques. Polyphenols reverse this dysbiosis by increasing beneficial bacteria such as Akkermansia muciniphila, Lactobacillus, and Bifidobacterium.	The microbiota modified by polyphenols modulates the secretion of key neurotransmitters (increases GABA and serotonin, decreases glutamate excitotoxicity).	Polyphenols such as EGCG, curcumin, and resveratrol constitute a great therapeutic alternative thanks to their antioxidant effect and their ability to positively modulate the altered microbiota in ALS.	Moderate
**[57]**	Review	N/A (Review)	ALS (highlights hypermetabolism, weight loss, dysphagia, and severe gastrointestinal dysfunction).	Review on Dysbiosis and Fecal Microbiota Transplantation (FMT).	Mediterranean Diet, antioxidant vitamins (E, C, D), Omega-3 fatty acids, ketogenic diets, and clinical guidelines (DXA, BIA, energy expenditure equations).	Anorexia induced by psychosocial stress and depression.	Dysbiosis (alterations in the Firmicutes/Bacteroidetes ratio and low amounts of butyrate-producing bacteria) and an increase in neurotoxic bacteria that cross the compromised epithelial barrier.	Malnutrition and weight loss at diagnosis independently predict shorter survival. Alterations in lipid metabolism directly impact the prognosis.	Clinical management of ALS requires personalized nutritional strategies along with microbiota modulation tools (diets, FMT, probiotics) to mitigate	Low

									neuroinflammation and slow the disease.	
**[60]**	Review	N/A (Review)	Sporadic ALS and its relationship with brain development and the gut-brain axis in the first 1000 days of life.	N/A (Revisión)	Maternal diet, folate, solid diet after weaning, iron, and omega-3 fatty acids.	Maternal stress during pregnancy.	Unstable neonatal dysbiosis can persist and affect immunological and neurological development.	Defective microbiota maturation can trigger chronic inflammation and an alteration of the excitation/inhibition balance.	Microbiome maturation is considered an early environmental contributor in the "multi-step" hypothesis of ALS, opening pathways for early interventions.	Low
**[42]**	Randomized, double-blind, placebo-controlled trial	27 (14 FMT, 13 placebo).	Sporadic ALS (<2 years evolution, FVC >70%). ALSFRS-R, modified Norris scale, MiToS.	16S rRNA gene sequencing (V3 and V4) in fecal samples.	Intervention: Fecal Microbiota Transplantation (FMT). BMI control.	Evaluated using HAMD (depression), HAMA (anxiety), MMSE (cognition), and FSS (fatigue).	Significant increase in Bifidobacterium abundance at week 15 in the FMT group. Firmicutes and Actinobacteriota were prevalent.	FMT did not improve the ALSFRS-R score compared to placebo, but the FMT group showed statistically significant improvements in symptoms of depression, anxiety, and	Fecal microbiota transplantation is safe in ALS and improves concomitant non-motor functions (psychiatric and gastrointestinal), with a key increase	Low

								chronic constipation.	in Bifidobacterium.	
**[46]**	Observational case-control study	28 (16 ALS, 12 healthy controls).	Early-onset ALS (<6-15 months from symptom onset). 11 spinal onset, 5 bulbar onset. ALS-FR scale.	16S rRNA gene sequencing (V3 and V4) in feces.	Concentration of Short-Chain Fatty Acids (SCFA: acetic, propionic, butyric) evaluated by HPLC.	Not reported.	No changes in alpha-diversity. Increase in Fusobacteria, Acidobacteria, Enterobacter, and Veillonella. Decrease in Prevotella, Lactobacillus, and Butyricimonas.	Spinal-onset ALS patients showed a trend toward lower propionate content. The bulbar or spinal subtype influenced bacterial abundance.	The study demonstrates enteral microbial differences at the onset of ALS and highlights the significant heterogeneity in microbiome restrictions according to the disease subtype.	Low
**[26]**	Chemical/biological mechanism review	N/A (Review)	Cellular damage due to oxidative stress and its implication in diseases such as ALS and the use of specific drugs.	N/A (Review focused on metabolites)	Folic acid (vitamin B9) and its photoproduct, 6-formylpterin (6-FPT).	N/A	N/A (Focuses on folate photoproducts and cellular ROS generation).	6-FPT can generate reactive oxygen species (ROS) and has the ability to form covalent adducts with edaravone, a ROS-scavenging drug used for the treatment of ALS.	The intrinsic chemical reactivity of 6-FPT must be strongly considered, especially the monitoring of adduct formation in ALS patients undergoing chronic	Moderate

									treatment with edaravone.	
**[61]**	Prospective longitudinal cohort and bioinformatics	N/A (Review)	Life-course exposome risk factors and clinical heterogeneity (bulbar vs. spinal onset).	Literature review on microbiome alteration (sequencing and associated metabolomics).	Energy intake, body mass index (BMI), lipids, fiber, ketogenic diet intervention, and supplements.	Cognitive performance, socioeconomic status, and lifestyle habits (smoking, alcohol).	Lower levels of butyrate-producing bacteria are reported in ALS. The microbiome correlates with disease progression and the site of symptom onset.	There are potential shared causal effects between environmental exposures (metals, pesticides, diet, altered microbiome) and a polygenic predisposition in ALS (gene-time-environment hypothesis).	Prospectively understanding the ALS exposome (including microbiota and diet) and gene-environment interactions could offer prevention perspectives and identify rational interventions.	Low
**[20]**	Prospective longitudinal cohort and bioinformatics	185 (75 ALS, 110 controls). Metabolic overlap: 54 ALS, 79 controls.	ALS evaluated with ALSFRS-R (median 37-38). Differentiated between bulbar, cervical, or lumbar onset.	16S rRNA gene sequencing (V4) in fecal samples and PCoA.	Non-targeted plasma metabolomics identified lipid profiles (acylcarnitines, fatty acids, bile acids). BMI and energy metabolism data.	Not reported.	Decreased alpha-diversity in ALS. Modification in the abundance of Firmicutes, Cyanobacteria, and Bacteroidetes. Decrease in butyrate-producing bacteria, Faecalibacterium, and Bifidobacterium.	Several strains (ASVs), particularly from the Lachnospiraceae family, strongly inter-correlated with plasma lipid metabolites. The increase in acylcarnitines correlated with	The gut microbiome differs structurally in ALS and is closely correlated with plasma lipid profiles, providing a biomarker link and	Low

							the decline in the ALSFRS-R scale.	mechanistic information through the microbiome-metabolome network.	
**[11]**	Cross-sectional observational study (spouse-controlled)	40 participants (10 ALS patients, 10 spouse controls, and 20 healthy paired volunteers).	Recent diagnosis (<2 years) of sporadic ALS. Mean ALSFRS-R of 39.9.	16S rRNA gene sequencing (V4 region) from rectal swabs.	Diet/environment control by living under the same roof. Fiber intake not evaluated in detail, but microbial metabolism was assessed.	Consideration and attempt to rule out psychological stress in couples as a confounding variable.	Higher microbial diversity (alpha-diversity) in ALS patients compared to spouses. Marked deficiency of the genus Prevotella spp. (specifically P. timonensis).	Levels of plasma or fecal inflammatory markers (cytokines, LPS) showed no significant differences between ALS patients and their spouses.	By eliminating environmental variables shared with partners, it is observed that microbial composition is fundamentally distinct in ALS; Prevotella deficiency and altered butyrate metabolism offer therapeutic potential.	Low
**[71]**	Review of GBA interventions	N/A (Review)	Impact of neuroinflammation, excitotoxicity, and decreased gastrointestinal	Review of alteration, translocation, and abundance changes in	Ketogenic diets, SCFA/AGCC (such as butyrate) derived from	Regulation of the Hypothalamic-Pituitary-Adrenal	Multiple studies report a loss of protective SCFA-producing bacteria and enrichment of endotoxin-producing (LPS) pathogens,	"Leaky gut," triggered by dysbiosis, allows the systemic entry of toxic and	The microbiome bidirectionally regulates the central nervous	Low

			tract motility in ALS.	intestinal ecosystems.	fiber, polyphenols, and the use of probiotics.	(HPA) axis, stress response hormones, and mood mediated by intestinal neurotrans mitters.	affecting the intestinal barrier.	inflammatory metabolites (LPS) and promotes abnormal aggregation and degeneration.	system through the GBA. Modular therapies (FMT, probiotics, bacteriophag es, and diets) represent a very promising new era in the treatment of ALS.	
**[80]**	System atic / Narrati ve Review of ALS Pathog enesis	N/A (Review)	Degeneration of upper and lower motor neurons, oxidative stress, and muscle atrophy.	Theoretical analysis of bacterial community imbalance and intestinal pathogens.	Dietary habits and immunomodul atory metabolic production (SCFAs).	Circadian rhythms and psychologic al states that modulate the microbiota and influence the GBA Axis.	The imbalance in the Firmicutes/Bacteroidetes ratio alters SCFA production. Production of neuroinflammatory factors that activate astrocytes and microglia (glial cells).	Dysbiosis and extracellular vesicles (EVs) foster systemic inflammation through the crossing and permeability of the blood-brain barrier, inducing motor neuron death.	Identifying and modulating targets related to genetic variants, intestinal dysbiosis, extracellular vesicles, and excessive glial activation offers the best hope and direction for the	Low/M oderat e

									treatment ("Cocktail Therapy") of ALS.	
**[82]**	Review	N/A (Review)	Patients with major depressive disorder, including treatment-resistant cases. Does not mention ALS.	Fecal bacterial composition analysis using 16S rRNA gene sequencing and LC/MS in the cited studies.	Impact of Western and "fast-food" diets on the reduction of bacterial diversity. Use of omega-3 and prebiotics.	Evaluation of depressive symptoms, anxiety, obsessive behavior, and quality of life.	Dysbiosis characterized by a decrease in Lactobacillus and Bifidobacterium, and an increase in Bacteroides and Proteobacteria.	Correlation between antibiotic use and risk of depression. Association between cytokine levels (IL-6, IL-1beta, CRP) and clinical severity.	Neuroinflammation and dysbiosis are pillars in the etiology of depression. "Fecomodulation" treatments (probiotics and FMT) are promising.	Low
**[49]**	Narrative Review	N/A (Review)	Fatal disease with damage to upper and lower motor neurons, muscle weakness, paralysis, spasticity, and respiratory failure.	N/A (Review)	Diets (high-fat, hypercaloric, ketogenic), polyphenols, flavonoids (myricetin), vitamins (D, E, B12, C, A, K), prebiotics, and probiotics.	Modulation of neurotransmitters (GABA, serotonin) crucial for stress, cognitive function, and emotional state.	Decrease in microbial diversity, alteration of the Firmicutes/Bacteroidetes ratio, and a sharp drop in SCFA producers such as Faecalibacterium prausnitzii, Eubacterium rectale, and Roseburia intestinalis.	The alteration of the Firmicutes/Bacteroidetes ratio and the translocation of bacteria into the blood correlate strongly with the severity of clinical symptoms (ALSFRS-R scale).	The modulation of the microbiota through dietary components, probiotics, and fecal transplantation is a promising supportive strategy to reduce neuroinflam	Low

									mation in ALS.	
**[69]**	Review	N/A (Review)	Irreversible motor impairment, bulbar weakness, dysphagia, sialorrhea, constipation, and delayed gastric emptying.	N/A (Review)	Glutamate, red/processed meats, sodium, and fats increase risk. Coffee, tea, and vegetables are protective. Modulation by short-chain fatty acids (SCFAs), prebiotics, and probiotics.	Subtle cognitive impairment and 5-15% overlap with overt Frontotemporal Dementia (FTD).	Decrease in butyrate producers (*Butyrivibrio fibrisolvens*, *Clostridium spp.*, *Roseburia*); increase in *Odoribacter*, *Parabacteroides*, *E. coli*, and Enterobacteria.	Disease severity in patients correlates directly with gastrointestinal symptoms such as dysphagia and chronic constipation.	The gut-brain axis is altered, and the use of interventions targeting the microbiota (FMT, probiotics) has great potential to attenuate progression and mitigate excitotoxicity.	Low
**[66]**	Review	N/A (Review)	Damage to upper and lower motor neurons, muscle wasting, adult-onset paralysis, and respiratory failure.	N/A (Revisión).	Fecal Microbiota Transplantation (FMT) and synthesis of SCFAs (acetate, propionate, and butyrate) in the gut.	Cognitive and behavioral changes (poor memory, apathy, irritability) and high stress and emotional agitation in family caregivers.	Reduction of butyrate-producing bacteria (*Eubacterium rectale*, *Roseburia spp.*), lower abundance of *Succinivibrionaceae*, and enrichment of pro-inflammatory taxa.	Changes in the Firmicutes/Bacteroidetes ratio are associated with greater disease severity and shorter survival.	Fecal Microbiota Transplantation (FMT) represents a promising experimental therapeutic strategy that complements current treatments by targeting the gut-brain axis.	Low

**[50]**	Comprehensive Review	N/A (Review)	Loss of motor neurons, spasticity, weakness, atrophy; significant clinical and protein (TDP-43) overlap with frontotemporal dementia.	N/A (Review)	Nicotinamide, prebiotic supplements (polyphenols, inulin, GOS, FOS, resistant starch), probiotics, and FMT.	Profound non-motor cognitive involvement; up to 50% exhibit cognitive/behavioral impairment and frontal/temporal lobe deterioration (pseudobulbar affect).	Critical role of *Akkermansia muciniphila*, *Ruminococcus torques*, *Butyrivibrio fibrisolvens*, and *Clostridium tyrobutyricum* in the course of ALS dysbiosis.	ALS patients have significantly lower nicotinamide levels in serum/cerebrospinal fluid. The microbiome of bulbar or spinal onset exacerbates symptoms.	Future focus is on personalized medicine, combining gene editing therapies (ASO, RNAi, CRISPR) with microbiome-modulating interventions (FMT/probiotics).	Moderate
**[52]**	Clinical / Pharmacological Review	N/A (Review)	Degeneration of the central and peripheral nervous system, cramps, fasciculations, asthenia, sialorrhea, spasticity, musculoskeletal pain.	N/A (Focus on treatments)	Hypercaloric therapy, monitoring of polyunsaturated fatty acids, vitamins (B12, E, B6, niacin, folate, selenium), pre/probiotics, antibiotics, and FMT.	Correction of anxiety, depression, sleep disturbances, and emotional-volitional insufficiency (palliative psychology).	Microbiome normalization is highlighted as a potential target to be corrected through probiotics, diets, and fecal microbiota transplantation (FMT).	N/A (Generic pharmacology review; however, notes that high lipid levels could correlate with longer survival).	Approved pathogenetic treatments (riluzole, edaravone) have low efficacy. New pathways (immunomodulators, stem cells, and microbiome therapies) are being investigated for prevention.	Low

**[51]**	Review	N/A (Review)	Progressive atrophy, muscle weakness, fasciculations, spastic hypertonia, and hyperreflexia. Bulbar or spinal involvement.	N/A (Review)	Investigated antioxidant treatments (vitamins E and C, carotenes, flavonoids, resveratrol, turmeric, melatonin). Digestive alterations change the diet.	Bulbar involvement is highly associated with cognitive impairment (10% frontotemporal dementia), emotional alteration, and depression.	Dysbiosis revealed as increases in *Anaerostipes hadrus*, *Bacteroidales*, *Bifidobacterium pseudocatenulatum*, and decreases in *Clostridium leptum*, *E. coli*, and *Akkermansia muciniphila*.	It was observed that a longer intestinal transit time in the animal model coincided temporally with the onset of limb weakness.	Oxidative stress and microbiota alterations affecting the enteric nervous system (gut-brain axis) are key conditioning and pathogenic factors to halt in ALS.	Moderate
**[43]**	RCT Protocol (FETR-ALS)	42 (28 planned in the FMT group and 14 in the placebo control group).	Early-stage patients (symptom onset no longer than 18 months); familial or sporadic ALS.	(Planned): Exhaustive compositional analysis of the microbiome in saliva, intestinal tissue, and fecal samples over multiple months.	Primary intervention with allogeneic infusion of healthy feces (Fecal Microbiota Transplantation - FMT) via nasojejunal tube or endoscopy.	Specific assessment questionnaire for Quality of Life in Amyotrophic Lateral Sclerosis (ALSAQ40) at 6 and 12 months.	(Based on reports): A significant reduction of beneficial populations (*Oscillibacter*, *Anaerostipes*) and an increase in harmful ones (*Dorea*) was previously mentioned.	(Hypothesis): FMT will increase regulatory T cells (Tregs) by 20%, transforming the immune environment surrounding motor neurons into an anti-inflammatory state.	It is the first trial designed to evaluate whether Fecal Microbiota Transplantation can act directly by modifying the neuroimmunological response and slowing the progression of early-stage ALS.	Low

**[68]**	Review	N/A (Review)	Sporadic ALS.	N/A	Smoking, diet, heavy metal toxicity, and environmental pollutants.	N/A	Tobacco smoke alters the microbiome (dysbiosis) and induces oxidative stress, promoting neuroinflammation.	Blood-spinal cord barrier (BSCB) dysfunction, exacerbated by dysbiosis and smoking, allows the influx of toxic immune cells toward motor neurons.	Smoking and microbiome alterations contribute directly and indirectly to ALS pathogenesis by creating and maintaining an environment of neurotoxicity.	Low
**[47]**	Longitudinal cohort with dietary intervention phase	84 (44 ALS, 40 controls). Follow-ups at T1 (n=36) and T2 (n=30).	Diagnosis <12 months. Evaluated with total ALSFRS-R, bulbar, respiratory, fine, and gross motor subscores.	LC-MS/MS to quantify Short-Chain Fatty Acids (SCFAs) in plasma.	Mediterranean Diet (6-month intervention).	Beck Depression Inventory II (BDI-II).	Baseline increase in acetic, propanoic, butyric, and 3-OH-butyric acid in ALS compared to controls; the diet significantly reduced acetic and propanoic acid.	Acetic acid correlated positively with depression (Beck) and negatively with fine motor function. 3-OH-butyric acid correlated negatively with total ALSFRS-R.	The Mediterranean Diet significantly modulates SCFA production in ALS, indirectly impacting motor function symptoms and depressive mood.	Low/Moderate
**[59]**	Review	N/A (Review)	Cognitive dysfunction,	N/A (Review)	Ketogenic Diet (KD),	Frontotemporal	The Ketogenic Diet alters microbial	Ketone bodies provide direct	Clinical management	Low

			behavioral changes, atrophy, and gradual muscle weakness.		hypercaloric supplements, vitamins (B1-12, A, C, E), probiotics, prebiotics (GOS, FOS), and Fecal Microbiota Transplantation (FMT).	dementia, cognitive impairment, depression, and anxiety.	composition (increasing *Akkermansia* and *Lactobacillus*), but its low carbohydrate content reduces global diversity and SCFA production.	energy and reduce the NLRP3 inflammasome. Prebiotics (GOS) attenuate neuroinflammation and reverse cognitive damage.	of ALS requires a multidisciplinary approach. The ketogenic diet and microbiome modulation with synbiotics are promising therapies that demand more human trials.	
**[41]**	Case-control study	139 (66 ALS patients, 61 healthy controls, 12 neurodegenerative controls).	Familial and sporadic ALS. ALSFRS-R (median 36) and respiratory vital capacity.	Shotgun metagenomic sequencing and 16S rRNA in fecal samples.	Use of gastrostomy tube recorded as a clinical variable (15.2% of cases).	Wexner Constipation Scale (GI function and discomfort).	Significant decrease in dominant butyrate-producing bacteria (*Eubacterium rectale*, *Roseburia intestinalis*, *Bifidobacterium longum*) in ALS patients compared to controls.	The Wexner constipation score correlated with the abundance of 7 species. No species significantly correlated with the progression of the ALSFRS-R scale.	Butyrate-producing bacteria (key for inflammatory regulation) are reduced in ALS, highlighting the microbiome as a risk factor and potential therapeutic target.	Low

**[72]**	Review	N/A (Review)	Motor neuron degeneration and a broad range of motor and non-motor (gastrointestinal)	N/A (Review)	Nutrient absorption, metabolic impact of enteral diet, and bacterial metabolism of medications such as Riluzole.	Depression and anxiety.	High inter-individual variability; frequent but inconsistent reduction in the Firmicutes/Bacteroidetes ratio. Central impact of *Akkermansia muciniphila* and diversity reduction in late stages.	The microbiota modifies the pharmacokinetics of Riluzole and produces systemic metabolites (nicotinamide, SCFAs) that alter the immune response of the microglia (gut-brain axis).	The microbiota acts as a biological modifier. Integrating microbiome modulation and host genomics opens a real path toward precision medicine therapies in ALS.	Low
**[56]**	Narrative Review	N/A (Review of 125 reports/articles)	Gastrointestinal motility disorders such as severe gastroparesis and constipation, in addition to neurodegeneration.	N/A (Review)	Plant Bioactive Molecules (BPMs) and Essential Oils (EOs) (e.g., carvacrol, thymol, limonene, anethole).	Anxiolytic and antidepressant effects achieved through interactions with the GABAergic pathway using EOs	Clear dysbiosis with a reduction in beneficial strains (*A. muciniphila*, *Faecalibacterium prausnitzii*) and an increase in toxin/LPS producers (*E. coli*, *Bacteroides*).	Essential oils reduce intestinal pathogens, elevate antioxidant enzymes, and decrease neuroinflammatory mediators (IL-6, TNF-α), protecting the blood-brain barrier and the intestinal wall.	Supplementation with essential oils and BPMs offers a highly promising natural and complementary approach to regulate dysbiosis, gastric motility, and oxidative stress in ALS.	Moderate

**[65]**	Review (3 clinical trials and ongoing protocols)	N/A (Review of studies: n=1, n=2, and n=27 humans).	Sialorrhea, respiratory failure, progressive weakness, and paralysis. ALSFRS-R and ALSAQ-40 scales.	Metagenomic sequencing of the microbiome evaluated in the original reports analyzed.	Fecal Microbiota Transplantation (FMT) and Washed Microbiota Transplantation (WMT). Conventional use of Riluzole.	ALSAQ-40 quality of life assessment questionnaire and symptoms of anxiety/depression.	FMT successfully transplants the ecosystem toward that of the healthy donor, beneficially altering the intestinal metabolome (amino acid pathways such as arginine biosynthesis).	In two small reports (n=1, n=2), FMT halted deterioration, improved respiratory function, and muscle strength. In the largest controlled trial (n=27), there was no reduction in disease progression.	The divergence of results between studies highlights that FMT is feasible and hopeful but requires methodological refinement; antibiotic emptying prior to FMT could be counterproductive and nullify its effect.	Low
**[76]**	Preclinical animal model study	*C. elegans* ALS transgenic model (sod-1 A4VM) and controls (n=15 per experiment)	Oxidative stress model inducing specific motor neuron degeneration.	Intervention or exposure to probiotic strain *Enterococcus faecium* versus *E. coli*.	Probiotic *E. faecium* as food/intervention.	N/A	Exposure to *E. faecium* strongly induces the expression of genes linked to cytochrome P450 (CYP) oxidoreductases in the host.	*E. faecium* reduced intracellular ROS levels by activating the NHR-86 axis, significantly mitigating motor degeneration.	Intestinal activation of the NHR-86–CYP axis by *E. faecium* provides direct neuroprotection that improves	Low

									resilience in ALS models.	
**[75]**	Preclinical experimental study using non-targeted quantitative proteomics (TMT for the intestine and DIA for the liver).	N = 28 mice in total (7 mice per group: genotype/sex)	SOD1G93A transgenic mice (familial ALS model) and Wild-Type (WT) controls at postnatal day 120 (symptomatic stage).	Direct evaluation not performed; previous literature indicating dysbiosis in this model is cited.	Abundance of nutrient transporters was analyzed, finding an increase in glucose (SLC5A1) and cholesterol (NPC1L1) transporters specifically in males.	N/A	Not a primary finding, but the study discusses how "altered intestinal physiology" may relate to drug absorption.	Changes are highly **sex-dependent**: males show alterations in 140 hepatic proteins compared to only 3 in females.	Specific changes were identified in males that alter drug bioavailability (e.g., decrease in the CES1 enzyme), suggesting that ALS dosing should consider these peripheral changes.	Low
**[73]**	Review (Theoretical hypothesis)	N/A (Literature review)	Intestinal barrier dysfunction, progressive loss of motor neurons, neurotoxic inflammation.	Review of characterized dysbiosis in patients and models (diversity reduction).	Bacteria-derived metabolites: SCFAs (butyrate), nicotinamide, and precursors.	N/A	A selective loss of *Akkermansia muciniphila*, *Bifidobacterium longum,* and *Lactobacillus reuteri* causes a drop in systemic protective metabolites.	Deficiency in butyrate, nicotinamide, and microbial neurotransmitters (GABA) triggers a pro-inflammatory and excitotoxic cascade in the CNS,	Recontextualizes ALS as a gut-brain condition; supplementing metabolites or restoring key strains would stabilize neuroimmun	Low

								accelerating symptoms.	e homeostasis and slow the disease course.	
**[23]**	Review / Perspective.	N/A (Literature review)	Progressive muscular paralysis (familial and sporadic ALS) and gastrointestinal autonomic dysfunction.	Dysbiosis (bacteria, fungi, and viruses), with a focus on the reduction of butyrate-producing taxa and intestinal alteration.	Complex interaction between micronutrients (Vitamins B1, B12, D, E) and microbial metabolites such as butyrate, propionate, and TMAO.	Possible alteration in cognitive status in ALS derived from vitamin deficiency and dysbiosis.	Marked drop in diversity and beneficial SCFA-producing bacteria, independently correlated with intestinal barrier damage and systemic neuroinflammation.	The use of microbiota metabolites (phenylbutyrate) or high doses of specific vitamins (B12, D3) shows protective effects by slowing motor symptoms in trials.	Integrating therapies focused on correcting micronutrition (vitamins) and modulating the microbiota (probiotics, FMT) forms a path to improve patient survival.	Low
**[77]**	Systematic review of biological processes	N/A.	Age-associated neurodegenerative diseases (ALS, AD, PD, FTD); cortical atrophy, autophagy failure.	(Mentions systemic changes due to senescence in the gut-brain axis).	Use of probiotics and interventions for SCFA production in the gut.	Cognitive decline linked to senescent neuroinflammation, stress, and memory problems.	With aging, the balance of the gut microbiota is disrupted (dysbiosis), driving toxic cytokines (IL-6, TNF).	Modulating the gut-brain axis exhibits measurable improvement effects at the cognitive and global levels in elderly populations by combating neuroinflammation	The deterioration of vital processes due to aging triggers neurogeneration. Leveraging microbiome biomarkers is crucial in neuro-	Low

									geriatric precision medicine.	
**[78]**	In vitro and in vivo study (Preclinical)	NSC34-SOD1G93A cells and mutant SOD1G93A mice, with WT controls (n=8-11 per subgroup for behavior).	Rotarod test, neurological scoring of progressive paralysis, and survival curves.	N/A (Does not evaluate flora per se, but rather the direct mechanism of microbial SCFA metabolites).	Short-chain fatty acids (SCFAs) administered: sodium butyrate, sodium propionate, and sodium acetate.	N/A	N/A	Butyrate and propionate increased histone acetylation and induced mTOR-independent autophagy genes, degrading SOD1 aggregates and slightly extending survival.	Microbiota metabolites (SCFAs) improve survival by stimulating the autophagy of abnormal proteins, but the protective effect has a "ceiling" and disappears in the terminal phases of ALS.	Low
**[85]**	Bidirectional Mendelian Randomization (TSMR/MVMR)	Genetic prediction of neuropsychiatric disorders, including ALS.	Genetic prediction of neuropsychiatric disorders, including ALS.	Metabolic components measured by plasma lipid markers (e.g., phosphatidylcholine, triglycerides).	Metabolic components measured by plasma lipid markers (e.g., phosphatidylcholine, triglycerides).	Parallel risk evaluation for other disorders (Major Depressive Disorder - MDD, Schizophrenia, Bipolarity).	ALS risk genetically associated with the presence of *Sutterella*, *Bacteroides clarus*, and *Roseburia hominis*; and a decreased risk associated with *Dorea* unclassified.	8 lipids (e.g., various triglycerides) demonstrated risk causality toward ALS, while one type of phosphatidylcholine was protective. No reverse	Genetics validate specific causal relationships between the microbiome, lipid metabolism, and ALS, where certain lipids	Moderate

								causality was found (ALS does not primarily alter taxa in this model).	potentially act as intermediary factors (mediators).	
**[53]**	Epidemiological and nutritional review	N/A (Review)	Progression linked to hypermetabolism, loss of body mass, dysphagia, and oxidative stress.	Bibliographic analysis on the intestinal barrier and pre-asymptomatic microbial diversity.	Saturated dietary fats versus compounds like curcumin, creatine, CoQ10, vitamins (C, A, E), phytochemicals/polyphenols, and the neurotoxin BMAA.	Hypothalamic dysfunction, changes in eating behavior (preference for fats), appetite, and neuropeptide Y levels.	Early dysbiosis and an increase in pathogens alter circulating metabolites in the blood. Species such as *Akkermansia muciniphila* showed neuroprotective potential, while *Ruminococcus torques* worsens models.	Hypermetabolism induces compensatory higher caloric intake. Imbalanced diets and toxins like BMAA exacerbate the underlying cellular and motor damage of ALS.	There is a global discrepancy in ALS incidence linked to Western versus Eastern diets. Nutrition and microbiota correction dictate the neurodegenerative oxidative or toxic response.	Low
**[45]**	Clinical / Immunological review	N/A (Review)	Phenotypic heterogeneity in ALS (bulbar vs. spinal) evaluated via the ALSFRS-R scale; peripheral inflammation and *in*	Bibliographic examination of the microbiota-immunity axis.	Investigated systemic modulators: probiotics, Fecal Microbiota Transplantation (FMT), and intestinal	N/A.	Intestinal flora with a lower Firmicutes/Bacteroidetes ratio and low abundance of butyrate producers, which affects the activation of regulatory T cells (Tregs).	An association was found between intestinal barrier failure, circulating levels of inflammatory cytokines (IL-6, TNF),	Modifying and influencing the microbiota and the immune axis offers innovative approaches through personalized medicine	Moderate

			vivo neuroinflammation (PET).		butyrate production.			macrophages, and the severity of clinical scales regarding survival.	(immunomodulators and FMT) that are already underway in new trials.	
**[64]**	Systematic Review (Human and animal models)	N/A. Sum of 6 human microbiome studies (Total n=145 patients and n=152 healthy controls) and 6 animal studies.	Cross-sectional grouping of clinical data (including ALSFRS-R scales) with control for heterogeneous diagnosis (sporadic / paralysis).	Detailed analysis of the methodologies used (16S rRNA PCR versus shotgun metagenomics).	Production and direct role of Short-Chain Fatty Acids (SCFAs/Butyrate). Adverse effects of chronic use of PPIs (Proton Pump Inhibitors).	N/A.	Consistent decrease in butyrate-producing bacteria (*Anaerostipes*, *Lachnospiraceae*, *Eubacterium*, *Roseburia*). PPIs in the blood generate a chronic state that promotes the increase of *Streptococcaceae* families, affecting diversity.	Continued use of PPIs (gastric protectors) correlated with an increase in mortality (Hazard Ratio 1.34), possibly by aggravating previous dysbiosis. Butyrate promotes barrier resilience.	Divergences in previous studies are due to the use of low-resolution genetic techniques (16S). ALS requires shotgun metagenomics; the impact of PPIs and butyrate warrants preventive clinical surveillance.	Moderate
**[55]**	Clinical and physiological review	N/A (Literature review).	Non-motor peripheral symptoms: delayed gastric emptying, prolonged colonic transit times, incontinence,	Preclinical bacterial alteration (e.g., fALS models), evaluation of the F/B ratio, metagenomes, fecal studies	High-calorie diets to compensate for weight deficit, polyphenols (resveratrol/curcumin), probiotics,	Negative modulation of satiety hormones such as ghrelin or leptin, correlating with ALS-	Mice and humans show a progressive decrease in the anti-inflammatory species *Akkermansia muciniphila* and butyrate producers; significant increase in lipopolysaccharide	Damage to Paneth cells in the intestine decreases defensins, generating a "leaky gut" before the first central motor	Gastrointestinal involvements are routinely ignored despite preceding or accompanyin	Low

			dysphagia, and enteric nervous system denervation..	versus mouse colon/ileum.	nicotinamide, and future use of FMT transplantation.	induced anorexia.	(LPS)-induced cytokines and regional dysbiosis.	symptom; elevated LPS predicts fatal systemic inflammation.	g early stages; metabolically stabilizing the individual by optimizing the microbiota slows neurodegenerative pathways.	
**[58]**	Expert Critical Review	N/A (Review)	Marked weight loss, progressive resting hypermetabolism, and anorexia due to central damage, along with behavioral cognitive impairment.	Exhaustive comparative technical review on genetic analysis (16S rRNA versus metatranscriptomic and culturomic methods).	Alerts against pathogens that metabolize dietary precursors into toxins such as BMAA, TMA, and aromatic amines, as well as toxic metals (bismuth) and hydrocarbons (PAHs).	Gut-brain axis effects on mood, depression, and generalized anxiety, with a focus on the ALS and FTD overlap.	Reduction in overall phylogenetic diversity and abundance of *Ruminococcus* in ALS compared to controls; microorganisms process toxins but lose balance (eubiosis).	Dysbiosis and malabsorption promote wasting and aggravate lethal hypermetabolism; toxins formed in a leaky gut cross the blood-brain barrier, promoting the accumulation of protein aggregates.	Concludes that observed dysbiosis requires better control over dietary factors to avoid methodological biases and recommends using postbiotics (purified metabolites) as a safe	Low

								intervention for ALS.	
**[84]**	Cross-sectional case-control clinical study	50 total participants. Study group of 25 ALS patients and 25 healthy controls.	Diagnosis based on revised El Escorial criteria. Functional severity was assessed using the ALSFRS-R scale.	Analysis of alpha and beta diversity, along with taxonomic composition from phylum to genus level. Identification of biomarkers using the LEfSe method.	N/A	N/A	Significant reduction in alpha diversity in ALS patients.	Positive correlation between *Megamonas* abundance and ALSFRS-R scale scores. A decrease in short-chain fatty acids (SCFAs), specifically propionate and butyrate, was found in the ALS group.	Gut dysbiosis and SCFA reduction are implicated in ALS pathogenesis. Findings suggest that the microbiota and its metabolites could serve as diagnostic biomarkers and therapeutic targets.	Low
**[79]**	Systematic and narrative review article on the clinical impact of immun	N/A (Review)	Sporadic and familial ALS patients; discusses phenotypic heterogeneity and disease progression.	Analyzes gut dysbiosis as a factor preceding motor symptoms, increasing intestinal permeability and immune activation.	Mentions the influence of metabolism and diet on microbiota composition and its impact on neuroinflammation.	The study focuses on neuroinflammatory and clinical biomarkers rather than psychological variables.	Highlights the reduction of butyrate-producing bacteria and the increase in pro-inflammatory genera that exacerbate the systemic response.	Correlation found between high levels of cytokines (IL-6, TNF-alpha) and neurofilaments in CSF with faster disease progression.	The immune system plays a dual role (neuroprotective initially and neurotoxic later). Immune modulation is a critical therapeutic target.	Moderate

	ity in ALS										
**[44]**		Systematic Literature Review	N/A (Review)	Patients with communication capacity, FVC >70%, without nocturnal hypoventilation or prior mental illnesses. SOD1 G93A murine models.	16S rRNA gene sequencing and microbial diversity analysis through high-throughput sequencing.	Evaluation of nutritional status, weight loss, and dietary supplementation with 2% butyrate in water.	Emotional and cognitive health outcomes as potential results of the microbiota-gut-brain axis.	Reduction of butyrate-producing bacteria (*Butyrivibrio fibrisolvens*). Increase in levels of *Dorea* (a potentially harmful genus).	Association between dysbiosis and increased intestinal permeability (leaky gut). Butyrate delayed death in mice by 38 days.	There is "modest evidence" implicating the microbiota in the pathophysiology of ALS. Longitudinal studies are required to confirm causality.	Moderate

ADI-12= ALS Depression Inventory-12; ALSAQ-40= Amyotrophic Lateral Sclerosis Assessment Questionnaire-40; ASVs= Amplicon Sequence Variants; BAI= Beck Anxiety Inventory; BDI-II= Beck Depression Inventory-II; BIA= Bioelectrical Impedance Analysis; BMI= Body Mass Index; BPMs= Plant Bioactive Molecules; CSS= Constipation Scoring System; DXA= Dual-energy X-ray Absorptiometry; Eos= Essential Oils; FSS= Fatigue Severity Scale; GOS/FOS= Galacto-oligosaccharides / Fructo-oligosaccharides; HAMA= Hamilton Anxiety Rating Scale; HAMD= Hamilton Depression Rating Scale; HDAC= Histone Deacetylase; HPLC= High-Performance Liquid Chromatography; JBI= Joanna Briggs Institute; LC/MS= Liquid Chromatography-Mass Spectrometry; MMSE= Mini-Mental State Examination; PCoA= Principal Coordinates Analysis; PPI= Proton Pump Inhibitor; Tregs= Regulatory T cells.
